# Zoological transmission of encephalomyocarditis virus in the United States: Virus evolution, host ecology, and capsid antigenicity derived from an outbreak

**DOI:** 10.1371/journal.ppat.1013861

**Published:** 2026-02-17

**Authors:** Aastha Adhikari, Kurtis H. Feng, Ayusha Shrestha, Carlos E. Rodriguez, Elizabeth C. Nolan, Geoffrey W. Pye, Phillip C. Gauger, Thomas Denagamage, Edward C. Holmes, Andrew B. Allison

**Affiliations:** 1 Department of Comparative, Diagnostic, and Population Medicine, College of Veterinary Medicine, University of Florida, GainesvilleFlorida, United States of America; 2 Disney’s Animals, Science, and Environment, Bay Lake, Florida, United States of America; 3 Department of Veterinary Diagnostic and Production Animal Medicine, Veterinary Diagnostic Laboratory, College of Veterinary Medicine, Iowa State University, Ames, Iowa, United States of America; 4 Department of Large Animal Clinical Sciences, College of Veterinary Medicine, University of Florida, Gainesville, Florida, United States of America; 5 School of Medical Sciences, University of Sydney, Sydney, New South Wales, Australia; Lankenau Institute for Medical Research, UNITED STATES OF AMERICA

## Abstract

Encephalomyocarditis virus (EMCV) is a rodent-borne picornavirus that has repeatedly caused severe outbreaks resulting in the deaths of zoo mammals – most notably elephants – for decades. However, within North America, little is known regarding the diversity of EMCV that exists in nature, the reservoir or amplifying hosts important for maintaining the virus, and the epidemiology of zoo-associated outbreaks. This lack of knowledge of the EMCV strains that circulate in North America has impeded a more thorough understanding of how genetic and antigenic variation may affect pathogenicity or potentially vaccine-induced protection from disease. Herein, following a zoological outbreak of EMCV in Florida, we conducted the most comprehensive comparative phylogenomic analysis of virus isolates from fatal zoo animal cases and local rodent species to date, identifying both non-native, invasive rodents and native mice and rat species as potentially important in precipitating and/or maintaining outbreaks, with multiple transmissions to zoo animals. After development of an autogenous vaccine, we investigated the duration and magnitude of neutralizing antibody responses in elephants monthly for multiple years, providing a unique fine-scale, long-term profile of responses to vaccination. To better understand how antigenic variation may affect vaccine-induced protection, we constructed a reverse genetics system to determine the level of cross-protection afforded by autogenous vaccination against capsids derived from various divergent EMCV strains and serotypes. These results provide new advancements in understanding EMCV transmission and antigenicity in North America, which can be used as a foundation to ultimately enable zoos to better protect animals from this important pathogen.

## Author summary

Much has been learned about the molecular biology and pathogenesis of encephalomyocarditis virus (EMCV) over the last several decades, as it has been used as an important model system on many fronts, including deducing mechanisms of myocardial pathogenesis and viral-induced diabetes, antiviral immunity, polyprotein processing, as well as a prototype for protein expression via an internal ribosome entry site (IRES), ribosomal skipping, and ribosomal frameshifting. Despite this wealth of information, very little is known about the ecology and evolution of the virus in nature, in particular in the United States. To zoological institutions, EMCV is a major concern, as it can cause devastating outbreaks resulting in fatal myocarditis and/or encephalitis in numerous wildlife species. Herein, we investigated an outbreak that was precipitated by the death of an African elephant, in hopes to begin to shed light on the transmission dynamics and genetic and antigenic diversity of EMCV that exists in North America.

## Introduction

Encephalomyocarditis virus (EMCV) is a single-stranded, positive-sense RNA virus in the genus *Cardiovirus*, family *Picornaviridae*, that is classified as the species *Cardiovirus rueckerti* (formerly known as species *Cardiovirus A*) [1]. The genome is ~ 7.8kB in length and encodes a polyprotein that is subsequently cleaved into individual proteins (L-VP4-VP2-VP3-VP1-2A-2B-2C-3A-3B-3C-3D) primarily by the viral protease 3C^Pro^ [2]. Four of these proteins – VP1, VP2, VP3, and VP4 – assemble to form an icosahedral capsid ~30 nm in diameter, with the capsid containing 60 copies of each of the four proteins [3]. As VP4 resides internally, VP1, VP2, and VP3 constitute the outer surface of the capsid, with each of these three proteins composed of an eight-stranded, anti-parallel β-barrel that contains interconnecting loops of variable length which define the antigenicity of the capsid and, hence, the serotype of the virus [3,4]. Currently, there are two serotypes recognized by the International Committee on Taxonomy of Viruses, EMCV-1 (cardiovirus A1) and EMCV-2 (cardiovirus A2), the latter being first detected in 2005 [5].

From a zoological standpoint, EMCV (EMCV-1) was initially described in an infected gibbon (species not reported) and chimpanzee (*Pan troglodytes*) in 1944–45 that were housed at the Anthropoid Ape Research Foundation in Dania Beach, Florida, both of which died from peracute interstitial myocarditis and pulmonary edema [6]. Subsequent studies in laboratory mice demonstrated that the chimpanzee isolate induced hind limb paralysis (indicative of neurological involvement), in addition to acute myocarditis, leading to the derivation of its common name [6]. In 1946, a virus initially designated as Mengo encephalomyelitis virus (i.e., Mengo virus) was isolated from a captive rhesus macaque (*Macaca mulatta*) from the Mengo district of Buganda, Uganda, which had been euthanized after developing flaccid paralysis in both legs [7]. In subsequent serological testing, Mengo virus was shown to be antigenically indistinct from EMCV, suggesting the two were geographical variants of the same virus [7]. Mengo virus, along with other antigenically similar and historically identified rodent-associated cardioviruses – collectively known as the Columbia SK group of viruses – are now classified as EMCV [8,9]. Since these initial isolation and serological characterization studies, EMCV has been shown to have a global distribution [10].

EMCV is believed to be principally rodent-borne, being maintained in nature in wild rats and mice [10–12]. In experimental infection studies of Wistar rats (*Rattus norvegicus domesticus*), EMCV was excreted in feces for several weeks post-infection, with efficient horizontal rat-to-rat transmission and a lack of any clinical signs [13]. Although rodents are currently viewed as the likely reservoir hosts for EMCV worldwide, the exact rodent species that maintain the virus in nature are largely unknown and may differ between geographical regions, as recently shown with *Mastomys* rodents in southern Africa [12]. During surveillance studies in the United States, a number of common wildlife species other than rats and mice have been shown to be infected with EMCV, including other small mammals such as squirrels and rabbits [9,14–16]. Whether these infections represent spillovers from the local rodent reservoir, or that species other than rats and mice may be involved in the maintenance of the virus during non-epizootic periods, remains unclear. Additionally, wild boars have been suggested as potential reservoir species in Europe and as a source of infection for domesticated swine [17,18], while the role of feral swine in the epidemiology of the virus in the United States has not been evaluated. The virus has also been detected in birds, as well as arthropod vectors such as mosquitoes and ticks, although there is no evidence that the virus is bird-associated or arthropod-borne, and these detections are presumed to be the result of incidental infection or inadvertent blood meals from a viremic host [10]. Overall, the natural transmission cycles of EMCV in North America (and globally) are largely enigmatic.

In natural infections of susceptible mammalian species such as zoo animals or domestic swine, EMCV tends to affect the cardiopulmonary system, with additional neurological involvement in some cases. The major target organ is the heart, with clinical infection predominantly manifesting as myocarditis, with pulmonary edema often noted secondarily to cardiac failure [19–21]. EMCV outbreaks in zoos – involving a myriad of mammal species such as primates, ungulates, carnivores, rodents, and marsupials – have been reported from Australia, Asia, Africa, Europe, and North America, making the virus a worldwide problem in zoological settings [16,22–29]. Although EMCV is generally regarded as an animal pathogen, it is also zoonotic, with sporadic human infections that are generally inapparent or associated with mild to moderate symptoms such as fever, headache, malaise, abdominal pain, nausea, and/or vomiting [10,30]. To our knowledge, there have been no known reports of human deaths associated with EMCV infection.

Although fatal EMCV infections of free-ranging elephants have been previously documented, most notably an outbreak in Kruger National Park in South Africa in 1993–1994 that led to the death of 64 African elephants (*Loxodonta africana*) [31,32], knowledge on EMCV pathogenesis in elephants has been largely attained through zoo outbreaks [22,24,28,33]. Within the United States, EMCV infections in elephants have been predominantly reported from Florida, with the first authoritatively documented fatal EMCV case in an African elephant occurring in 1977 [22]. Between 1974–1978 in Florida, there were repeated outbreaks at three separate animal parks – Busch Gardens Tampa, Lion Country Safari West Palm Beach, and Jacksonville Zoo – in which 10 African and Asian elephants (*Elephas maximus*) were suspected or confirmed to have died from EMCV infection [22,23].

In 2019, we investigated an EMCV outbreak at a zoological institution in central Florida, where the index case was the peracute death of a 40-year-old female African elephant, which was followed by the deaths of three mandrills (*Mandrillus sphinx*), a North Sulawesi babirusa (*Babyrousa celebensis*), and a lion-tailed macaque (*Macaca silenus*), the latter an endangered species [34]. Deaths attributable to EMCV had not been previously experienced in the zoo’s history. In immediate response to EMCV being suspected as the cause of death of the elephant, susceptible animals were isolated from potential exposure to rodents (either by translocation of animals or creating rodent exclusions) and comprehensive rodent mitigation strategies were implemented (reducing harborage/food sources and significantly increasing existing integrated pest management efforts). Additionally, a vaccination program was initiated, first using an existing inactivated autogenous vaccine from another zoo, followed by the development of an autogenous vaccine derived from the index elephant case. With these actions, no further deaths attributable to EMCV occurred after the outbreak.

As the impacted zoo contains over 2,000 animals representing 300 + species, some of which are endangered (and potentially susceptible) mammal species, understanding the source and route of exposure that facilitated the outbreak, and how to better protect susceptible animals from disease, was of paramount concern. As such, we (i) conducted comprehensive phylogenomic and biological analyses of virus isolates from zoo animals and rodents to identify the natural hosts involved in the outbreak and examine the *in vitro* host range and fitness of naturally-circulating viruses, (ii) longitudinally investigated the duration and magnitude of neutralizing antibody responses to vaccination on a fine-scale by measuring titers on a monthly basis for multiple years, and (iii) constructed a reverse genetics system to determine the level of cross-protection afforded by autogenous vaccine-induced antibodies against various EMCV genotypes and serotypes found in North America and globally.

## Results

### Outbreak timeline, clinical signs, and pathology in zoological animals

**Fig 1:**
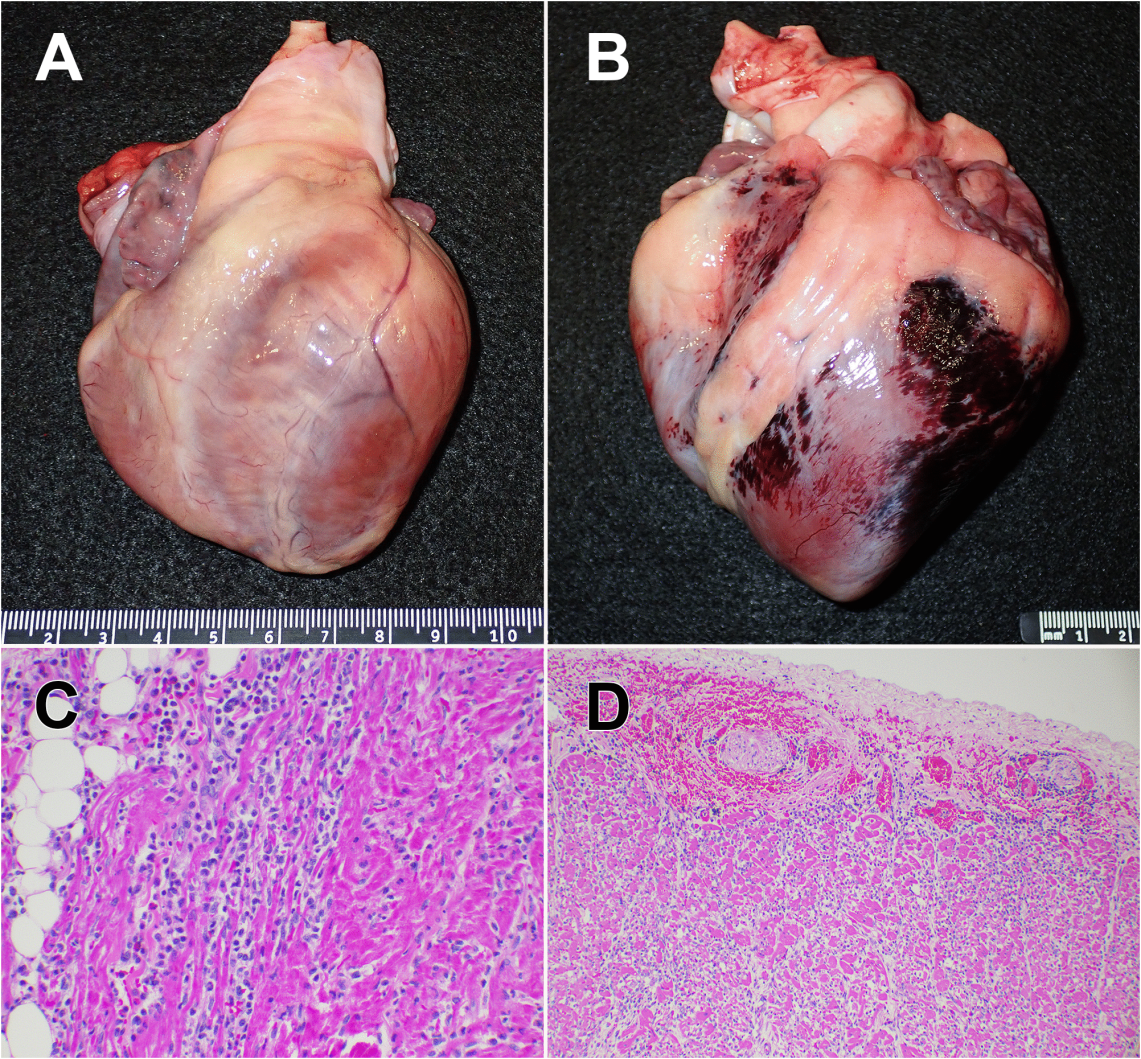
Clinical syndromes and associated gross and microscopic cardiac lesions noted in primate and non-primate zoo animals infected with EMCV during the outbreak. **(A)** Mandrill #3 heart: Clinical presentation of EMCV infection in primates – three mandrills and a lion-tailed macaque – was associated with intractable and progressive lethargy and dyspnea, decreased ventricular contractility, and signs consistent with congestive heart failure leading to death or euthanasia. Lesions at necropsy included pericardial, thoracic, and abdominal effusion, along with pulmonary edema. Note the pale streak-like areas on the heart (myocardial pallor) without epicardial or myocardial hemorrhages; **(B)** North Sulawesi babirusa heart: In contrast to the primate cases, gross lesions in the babirusa and African elephant centered on the heart and were characterized by broad, confluent areas of acute hemorrhage throughout the epicardium. Hemorrhages extended into the myocardium, interspersed with areas of myocardial pallor. Clinical presentation in both cases – babirusa and elephant – was sudden death with no, or limited and vague, premonitory signs; **(C)** Mandrill #3 heart: Patchy areas of myofiber degeneration and necrosis, with edema and mild to moderate neutrophilic and lymphocytic inflammation (20x magnification; hematoxylin and eosin [H&E] stain); **(D)** North Sulawesi babirusa heart: Hemorrhage along the epicardial surface and myocardium with myonecrosis, edema, and moderate neutrophilic and lymphocytic inflammation (10x magnification; H&E stain).

The index case of the outbreak was an African elephant. This was followed by the death of two mandrills, a North Sulawesi babirusa, another mandrill, and a lion-tailed macaque. The outbreak – defined here as the first to last zoo animal deaths – encompassed 26 days. The lesions in all of the zoo animals centered on the cardiovascular system with different patterns in the lesions observed in primate versus non-primate cases. The central nervous system, examined grossly and microscopically in all six cases, was unaffected. A clinical history and pathological assessment of each case is presented below for context:

(i) *African elephant* – 27 March 2019 – The 40-year-old, female elephant was found dead in a position that was highly suggestive of sudden death from a standing position. The previous day she was noted to be lame, but otherwise was eating, drinking, and acting normally. Upon necropsy, gross lesions were prominent in the heart, with broad areas of petechial hemorrhages and edema along the epicardial surface. There were foci of hemorrhage within the heart muscle, interspersed with patchy areas of pallor arranged in streak-like fashion throughout the width of the ventricular walls and septum. Histologic lesions observed in the heart included severe necrotizing myocarditis with sarcolemmal fragmentation, myofiber degeneration and necrosis, epimysial and perimysial edema, neutrophilic and lymphocytic inflammation, congestion, and epicardial hemorrhages.
(ii) *Mandrill #1* – 31 March 2019 – Four days after the death of the elephant, a mandrill (7-month-old, male) presented acutely lethargic after appearing normal behaviorally three hours earlier. Anesthesia was induced and he was noted to have a fast heart rate (tachycardia) with poor peripheral pulses, pale mucous membranes, and slow capillary refill times. Radiographs indicated an enlarged heart. Echocardiography and ultrasound showed a decrease in the ability of the heart to contract, with thin ventricular walls, sluggish blood flow, excessive free peritoneal fluid, and a possible blood clot in the portal vein. Cardiac arrest occurred shortly afterwards, and resuscitation was not successful. Gross lesions were prominent in both the heart and lungs. There was pericardial effusion and the epicardial surface of the heart, along with the cut surface of the ventricular free walls and interventricular septum, were mottled tan. The dorsal aspect of the lungs was dark red to purple (indicating congestion) and there was effusion in the abdominal cavity. Histologically, there was multifocal to coalescing, severe, acute necrotizing myocarditis with edema, hemorrhage, and mineralization.
(iii) *Mandrill #2* – 1 April 2019 – The second mandrill (2-year-old, female) was found acutely lethargic one day after the first mandrill. Following anesthesia, tachycardia, weak pulses, and white mucous membranes were observed prior to cardiac arrest and death. Upon necropsy, the epicardium and myocardium were diffusely pale, and the left ventricle was flaccid. Other gross and histologic lesions were similar to those observed in Mandrill #1.
(iv) *North Sulawesi babirusa* – 10 April 2019 – An 11-year-old, female babirusa was found recumbent in obvious distress and died very shortly afterwards. Upon necropsy, there were broad areas of hemorrhage and congestion throughout the epicardial surfaces of the heart (Fig 1B), along with pericardial effusion. On the cut surface, areas of epicardial hemorrhage correlated with areas of myocardial pallor with occasional intramuscular congestion and hemorrhage. Histologic lesions included acute, severe necrotizing myocarditis with sarcolemmal fragmentation, myofiber degeneration, epimysial and perimysial edema, multifocal intramuscular hemorrhages, neutrophilic and lymphocytic inflammation, and epicardial hemorrhages (Fig 1D).
(v) *Mandrill #3* – 10 April 2019 – The third mandrill (12-year-old, male) developed severe lethargy after being normal behaviorally two hours previously. Following anesthesia, tachycardia was noted, followed by a slow heart rate (bradycardia) and irregular rhythm, weak peripheral pulses, poor perfusion, and red and injected mucous membranes. On ultrasound, mild pericardial effusion, poor cardiac contractility of atria and ventricles, and uncoordinated muscle movement of the cardiac wall were noted. Euthanasia was performed due to the suspicion of EMCV infection. Grossly, there was peritoneal and pericardial effusion, with patchy to streak-like areas of myocardial pallor throughout the ventricular free walls of the heart, which was subjectively flaccid (Fig 1A). Histologic lesions included severe necrotizing myocarditis with sarcolemmal fragmentation, myofiber degeneration and necrosis, epimysial and perimysial edema, congestion and mild to moderate neutrophilic and lymphocytic inflammation (Fig 1C).
(vi) *Lion-tailed macaque* – 22 April 2019 – The 25-year-old, male lion-tailed macaque was found with acute lethargy, obtunded mentation, ataxia, and was minimally responsive to external stimuli. Behaviorally, he had been normal the night before. Following anesthesia, gray mucous membranes were noted prior to sudden cardiac arrest and death. Upon necropsy, there were multifocally extensive areas of epicardial and myocardial pallor throughout the ventricular free walls, with patchy to streak-like areas of pallor within the myocardium. Acute hemorrhage was minimal within the muscle wall, with limited pericardial effusion. Histologic lesions included severe necrotizing myocarditis with sarcolemmal fragmentation, myofiber degeneration and necrosis, epimysial and perimysial edema, and neutrophilic and lymphocytic inflammation.

### Virus isolation from zoo animals and rodents

For the six zoo animals that were found dead or were euthanized during the EMCV outbreak, virus was isolated from all heart samples (Ct values ranged from 15.0-21.8; > 40 negative) (S1 Fig), with characteristic cytopathic effects generally evident within 18–48 h of inoculation. During the 26-day outbreak period, EMCV was also isolated from trapped rodents within the zoo, marking (to our knowledge) the first time EMCV has been isolated concurrently from a local rodent population mid-outbreak. Of the 347 rodents collected during the outbreak period, 246 were tested for EMCV by qRT-PCR, of which 85 were positive (34.6%). A subset of 24 qRT-PCR positive intestine/kidney samples were tested for virus isolation, from which EMCV was recovered from nine individual rodents (37.5% of qRT-PCR positives) (S1 Fig), along with a novel adenovirus from a black rat (see below). Not surprisingly, the Ct values for the rodent samples were higher than those observed in the zoo animals, with rodent EMCV isolation-positive samples ranging from 28.5 to 36.6 (S1 Fig). Overall, virus isolation from qRT-PCR-positive rodents was inconsistent, although isolation was more likely to be successful with lower Ct values (S1 Fig). Positive RT-PCR results coupled with the absence of virus isolation has been noted previously with picornaviruses [35], and reported particle to plaque-forming unit (PFU) ratios of 250 ± 40 for EMCV [36] suggests that isolation from RT-PCR positive samples (e.g., with high Ct values/low levels of virus) may be unsuccessful due to such factors as lethal mutations or deletions in the genome, structural damage to the capsid, or that viruses are infectious, but fail to complete some step of the viral life cycle [4].

**Fig 2:**
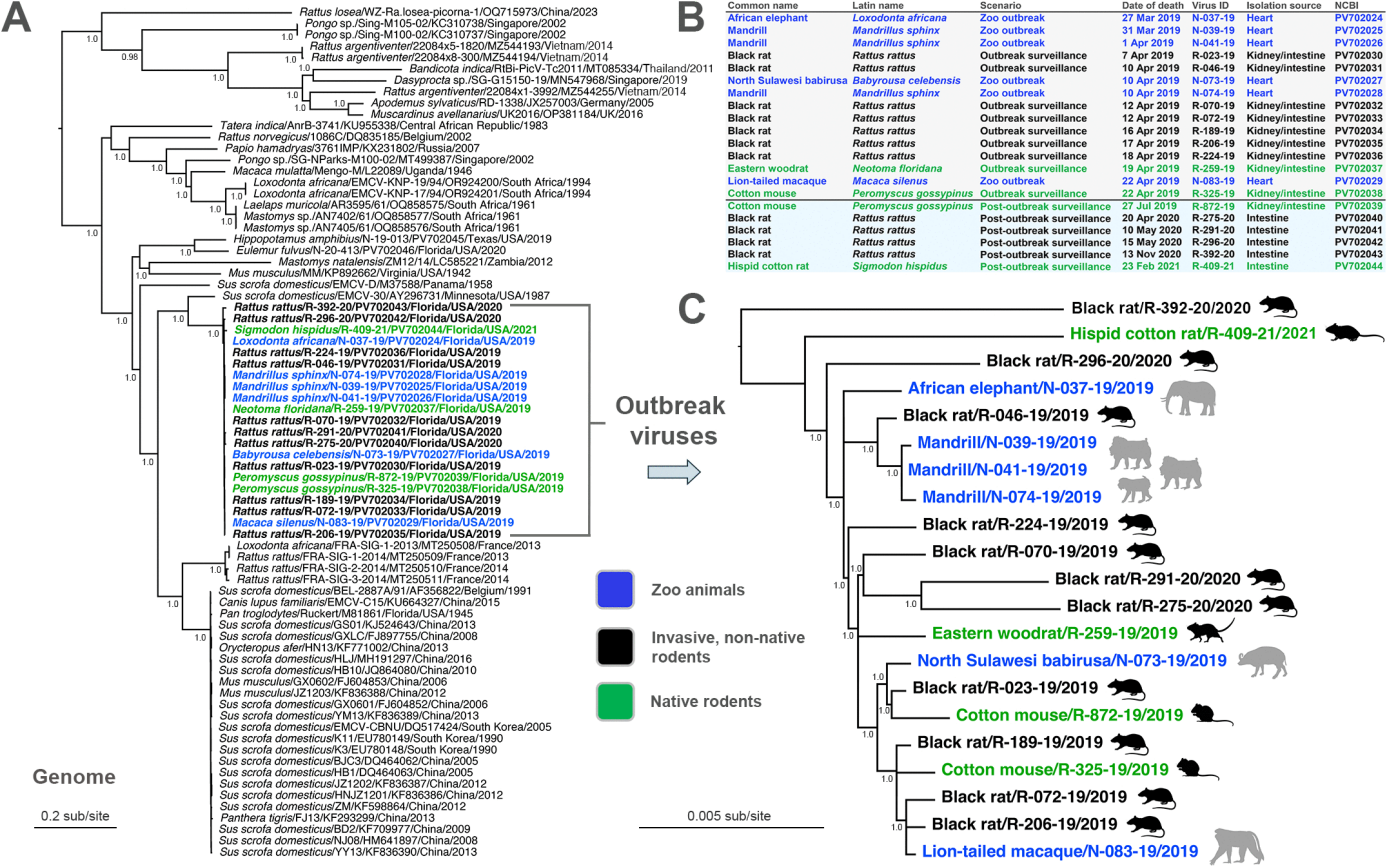
Evolutionary analysis of a zoological outbreak of EMCV using complete genomes from zoo animals and rodents. **(A)** A maximum likelihood phylogeny is shown based on complete genome nucleotide sequences of the 21 viruses isolated during the central Florida outbreak, along with a worldwide collection of EMCVs from Asia, Europe, Africa, and North America. For the 21 outbreak viruses, those recovered from zoo animals, invasive non-native rodents, and native rodents are shown in blue, black and green, respectively. The tree is mid-point rooted for clarity only. Horizontal branch lengths are scaled according to the number of nucleotide substitutions per site as shown, along with SH-like branch supports >0.9. Viruses are labeled as: species scientific name/ virus ID/ GenBank accession number/ state (where applicable)/ country/ detection or isolation date; **(B)** Summary of the 15 EMCV isolates recovered from zoo animals and rodents during the outbreak (27 March 2019 – 22 April 2019), in addition to 6 viruses isolated during post-outbreak rodent surveillance (27 July 2019 – 23 February 2021). Recovery of viruses is shown in chronological order by date of animal death (i.e., fatal zoo animal infection or rodent trapping). Virus isolate ID and source of tissue used for isolation and genomic sequencing are shown, with the concomitant NCBI accession numbers for each genome; **(C)** Maximum likelihood phylogeny of the complete genome nucleotide sequences of the 21 outbreak viruses in isolation. The tree is mid-point rooted for clarity only. Horizontal branch lengths are scaled according to the number of nucleotide substitutions per site as shown, along with SH-like branch supports >0.9. Viruses are labeled as: host common name/ virus ID/ isolation date and are color-coded as in panel A and B. Image was created, in part, using BioRender (Allison, A. [2026] https://BioRender.com/y37pu4t). Additional images were obtained from VectorStock [95].

Of the nine rodent isolations of EMCV during the outbreak, seven were from black rats (R*attus rattus*), one was from an eastern woodrat (*Neotoma floridana*), and one was from a cotton mouse (*Peromyscus gossypinus*), demonstrating that both invasive, non-native (black rat) and native (eastern woodrat, cotton mouse) rodents were simultaneously infected during the outbreak (Figs 2B and S1). During post-outbreak surveillance in the rodent population, we continued to isolate EMCV from intestinal samples of local rodents and, similar to the outbreak, viruses were recovered from both invasive, non-native (black rats) and native (cotton mouse and hispid cotton rat [*Sigmodon hispidus*]) rodents (Fig 2B). Between April 2020 and October 2020, we performed qRT-PCR and virus isolation on intestinal samples from 99 rodents, with EMCV detected by qRT-PCR in 16 rodents (16.2%), and from those samples, 3 viruses were isolated (18.8% of qRT-PCR positives). Lastly, from October 2020 to February 2021, we attempted virus isolation on intestinal samples (without correlative qRT-PCR testing) from 454 rodents, from which EMCV was isolated from a black rat in November 2020 and a hispid cotton rat in February 2021 (0.4%), the latter occurring almost two years post-outbreak.

In addition to EMCV, two other viruses were identified during rodent surveillance: (i) a novel adenovirus was isolated during the outbreak in April 2019 from pooled kidney and intestinal samples of an adult male black rat, tentatively named black rat adenovirus (genus *Mastadenovirus*, family *Adenoviridae*) (S1 and S2 Figs), and (ii) a mammalian orthoreovirus (MRV) (genus *Orthoreovirus*, family *Spinareoviridae*) was isolated from the intestines of an adult female hispid cotton rat during post-outbreak surveillance in February 2021 (S3 Fig).

### Genomic analysis of EMCV isolates

From the outbreak and post-outbreak surveillance, we sequenced the complete genome or near complete genome (see below) of 21 EMCV isolates from zoo animals and rodents. Additionally, we sequenced the genomes of two other isolates that were acquired from EMCV outbreaks in the southeastern United States during approximately the same time period: (i) N-19–013, isolated from a common (East African) hippopotamus (*Hippopotamus amphibius kiboko*) that died of necrotizing endomyocarditis at a zoo in Texas in 2018, and (ii) N-20–413, derived from a common brown lemur (*Eulemur fulvus*) that died from necrotizing panmyocarditis in 2020 at a zoological facility in south Florida. These two viruses were used in comparison to our outbreak viruses, in both phylogenetic analysis and neutralization testing.

Genetic comparison between the 21 genomes of deceased zoo animals and rodent viruses demonstrated that they shared a nucleotide identity between 98.3–99.9%. The genomes sequenced were from low passage cell culture isolates (passage 2 [P2] Vero E6), although the prototype elephant virus N-037–19 was additionally sequenced directly from heart tissue to determine the changes that might occur in culture. Comparison of the two genomes revealed only a single nucleotide change: an A to G transition at position 1129 resulting in a non-synonymous substitution of a threonine (ACT) to an alanine (GCT) at VP4 amino acid position 58 in the P2 cell culture passaged isolate. However, analysis of the RNA-Seq data demonstrated that this site was indeed polymorphic (i.e., contained a mixture of A and G, albeit with A more dominant) in elephant heart tissue, and Sanger sequencing of this site from a different heart sample from the elephant yielded a G at position 1129. Thus, while there is the potential for pre-existing mutations to be selected in cell culture, low passage isolates appear to be largely accurate representations of native genomic sequences. Genomes built from *de novo* assembly were then supplemented with 3’ RACE to verify the 3’ end of the genome from RNA-Seq data. However, 5’ RACE was unsuccessful, and in a number of cases, nucleotides mapping to the far terminal 5’ end were not resolved in the RNA-Seq data, leading to some genomes missing between 2–14 nt. As we performed cDNA synthesis for genome sequencing, the initial lengths of the polyC tracts in the 5’ UTR, which ranged from 14–25 nt, are inaccurate due to the inability of polymerases used in RNA-Seq to sequence long homopolymer stretches [37]. However, direct RNA sequencing of the prototype elephant virus N-037–19 (passage 2 BHK cell isolate) resulted in a longer polyC tract with a consensus sequence of 51 cytosines (5’-CAACCAAC_51_UCACUUU-3’). The maximum polyC tract observed was 57 cytosines, with each tract containing a few interspersed uridines without any discernable pattern to their placement. While this polyC tract is shorter than reported for EMCV isolates such as the prototype Rueckert strain (C_115_UCUCCCUC_10_) [38], it is on par with Mengo virus (C_50_UC_10_) [39]. However, as there is the potential for the length of the polyC tract to be artificially altered by cell culture passaging [37,40], future attempts at direct RNA sequencing should be made using fresh tissue when available. Attempts at extracting sufficient levels of high-quality polyadenylated RNA needed for direct RNA sequencing from archived frozen tissues from the remaining zoo animals were unsuccessful, which may have been a consequence of the small sizes of archived tissues, storage conditions (absence of RNA preservatives like RNA*later*), and/or length of storage (several years). Additionally, in the future, the lengths of the polyC tract determined by direct RNA sequencing versus RNase digestion and radiolabeling should be pursued for comparative studies.

### Evolutionary relationships of EMCV isolates

**Fig 3:**
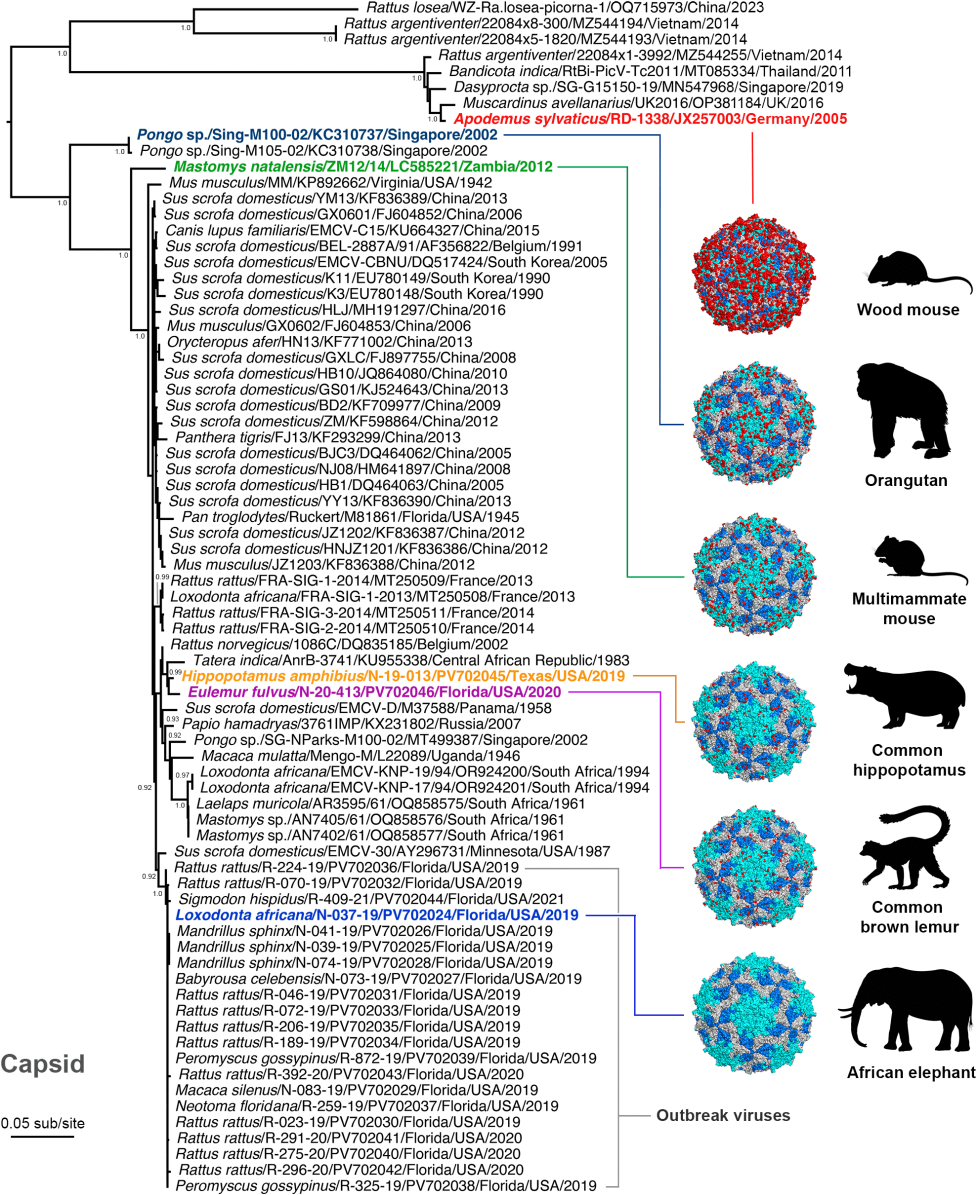
Maximum likelihood amino acid phylogeny based on capsid (VP4-VP2-VP3-VP1) sequences of EMCV. The capsid amino acid phylogeny was inferred using sequences of the 21 viruses isolated during the central Florida outbreak, along with EMCVs from Asia, Europe, Africa, and North America. In addition to the prototype outbreak elephant isolate (N-037-19), five other viruses analyzed in subsequent experiments (Figs 7 and 8) are highlighted in the tree: **(i)** EMCV N-20-413, isolated from a common brown lemur from a zoological facility in south Florida in 2020 (serotype 1), **(ii)** EMCV N-19-013, derived from an East African hippopotamus at a Texas zoo in 2018 (serotype 1), **(iii)** EMCV ZM12/14, isolated from a multimammate mouse in Zambia in 2012 (serotype 1), **(iv)** EMCV RD-1338, recovered from a wood mouse in Germany in 2005 (serotype 2), and **(v)** EMCV Sing-M100-02, isolated from an orangutan in Singapore in 2002 (tentatively designated as serotype 3). Nonsynonymous amino acid changes in the five viruses relative to N-037-19 were mapped onto the Mengo virus capsid crystal structure (pdb_00002mev) using PyMOL and are highlighted in red. The tree is mid-point rooted for clarity only. Horizontal branch lengths are scaled according to the number of amino acid substitutions per site. Only SH-like branch supports >0.9 are shown. Viruses are labeled as: host scientific name/ virus ID/ GenBank accession number/ state (where applicable)/ country/ detection or isolation date. Image was created, in part, using BioRender (Allison, A. [2026] https://BioRender.com/ej9nobp). Additional images were obtained from VectorStock [95].

**Fig 4:**
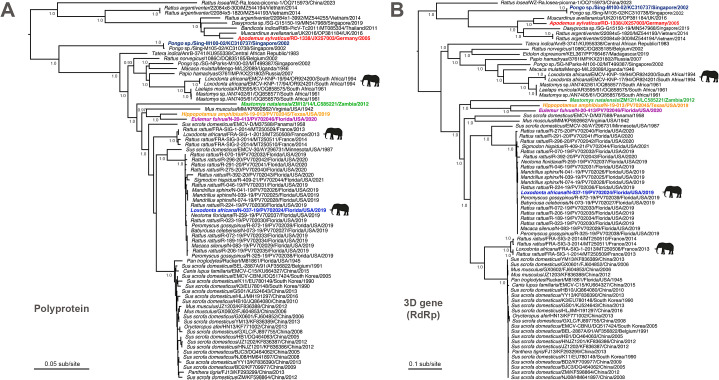
Maximum likelihood phylogenies based on complete polyprotein and 3D gene sequences of EMCV. **(A)** Polyprotein (amino acid) and **(B)** 3D (nucleotide) phylogenetic trees are shown. Three EMCV outbreaks involving elephants, in which sequences are available, are noted: two from zoological settings – Florida, USA in 2019 (this paper) and France in 2013 [28,43] – along with South Africa in 1994, in which 64 free-ranging elephants are thought to have died from EMCV infection [31,44]. Note the association of black rats (*Rattus rattus*) with both zoological outbreaks on separate continents. The six viruses noted in the capsid phylogeny (Fig 3) are also highlighted in both trees here. Although the topological position of the elephant/black rat French clade differs between the two trees, the associated SH-like branch support values are low. The trees are mid-point rooted for clarity only. The horizontal branch lengths are scaled according to the number of amino acid or nucleotide substitutions per site, respectively. Only SH-like branch supports >0.9 are shown. Viruses are labeled as: host scientific name/ virus ID/ GenBank accession number/ state (where applicable)/ country/ detection or isolation date. Image was created, in part, using BioRender (Allison, A. [2026] https://BioRender.com/ukh6rbu).

We first performed phylogenetic analysis with our 23 genomes (21 central Florida outbreak viruses, plus two viruses from separate outbreaks in Texas and south Florida) to determine their evolutionary relationships to each other and to other available EMCV sequences. A maximum likelihood phylogeny of complete EMCV genomes indicated that all central Florida outbreak viruses fell into a single clade with strong support, with the closest relative being isolate EMCV-30 recovered from a domestic swine in Minnesota in 1987 and which has been well studied as a pathogen [41,42] (Fig 2A). The phylogenetic grouping of the outbreak viruses with EMCV-30 was also observed in the capsid (Fig 3), polyprotein (Fig 4A), and 3D (Fig 4B) trees, reaffirming their close evolutionary relationship. While there is the potential for feral swine (which are abundant in Florida) to harbor EMCV [17,18], stringent biosecurity measures and regulations are in place within the zoo where the outbreak occurred regarding animal entry and transport on zoo grounds. In addition, during and around the time of the outbreak, no new domestic, feral, or wild swine entered onto the zoo property, and no feral swine were observed by personnel in the vicinity of the park. Collectively, this suggests that, while swine species were indeed involved in the outbreak (a fatal infection in one of the babirusas), the outbreak was most likely precipitated by the incursion of rodents into the park based on available data.

To examine the relationship among the central Florida outbreak viruses with higher resolution, we then analyzed the 21 genomes separately (Fig 2C). The rodent and zoo animal isolates were shown to intermix with each other in the phylogeny, forming well-supported sub-clades. This demonstrates efficient cross-species transmission of viruses not only between rodents and zoo animals as expected, but also between invasive non-native rats (i.e., black rats) and native rodents (e.g., cotton mice), which has not been previously shown. Notably, the rodent viruses fell in multiple locations within the outbreak phylogeny, indicative of genetic diversity as expected of a reservoir population, as well as multiple transmission events from rodents to zoo animals (rather than a single transfer event from a rodent to a zoo animal followed by repeated zoo animal-to-zoo animal transmissions) (Fig 2C). However, although the first and last mandrills died 11 days apart, the three mandrill viruses were very closely related and clustered together in a single clade (Fig 2C). This finding suggested that all the mandrills were infected by the same rodent virus (related to black rat isolate R-046–19) or possibly the mandrills may have spread the virus to each other, tentatively through normal social interactions (e.g., grooming) within the troop. While it may be presumed that most of the outbreak transmission events occurred from rodent-to-rodent, rodent-to-zoo animal, or possibly from zoo animal-to-zoo animal as with the mandrills, there is also the potential for virus transmission from zoo animals back into rodents. Ultimately, with many such scenarios in place, accurate tracking of the directionality of transmission during outbreaks is challenging. Similar to this epizootic, a previous outbreak in France also showed a similar phylogenetic relationship between isolates from black rats in association with the death of an elephant [43] (although note that while the phylogenetic position of the French outbreak clade differed between the polyprotein [Fig 4A] and 3D [Fig 4B] trees, SH-like branch support values were low at the key nodes).

While we isolated EMCV from a fatal case in a common brown lemur from south Florida in 2020, this virus was phylogenetically related to the hippopotamus isolate from Texas, rather than central Florida isolates in all trees (Figs 2A, 3, and 4A-4B), suggesting that these two viruses represent a previously unrecognized lineage that may be widespread within the southern United States. To further phylogenetically characterize various EMCVs (primarily EMCV-1), viruses have recently been grouped into eight lineages (designated A-H) and also subdivided into four major evolutionary clades (I-IV) [44]. Due to the probable existence of numerous lineages of EMCV that have yet to be discovered, we have refrained from using such alphabetical lineage/numerical clade terminology here for clarity.

In the capsid (VP4-VP2-VP3-VP1) phylogeny, EMCV-2 rodent isolates from Germany and the United Kingdom (JX257003 and OP381184) grouped with three Asian viruses (MZ544255, MT085334, MN547968) from *Rattus* and *Bandicota* rats and an agouti (*Dasyprocta* sp.) in the deepest lineage (Fig 3) [45,46]. As this grouping has strong support, and all these viruses share >97% amino acid identity, it suggests these viruses from Thailand, Singapore, and Vietnam are serotype 2 viruses. Similarly, other recently identified *Rattus* viruses from Vietnam (MZ544194, MZ544193) and China (OQ715973) [47] fell as a divergent sister clade to the EMCV-2 grouping (Fig 3), possibly representing one or two new serotypes as proposed (https://www.picornaviridae.com). The proposed novel serotype (EMCV-3) comprised of orangutan isolates (Sing-M100-02 and M105-02) from Singapore [27] was less divergent and clustered with the large EMCV-1 group in both capsid (Fig 3) and polyprotein (Fig 4A) phylogenies. However, within the genome and 3D phylogenies, these EMCV-3 isolates clustered with EMCV-2 and associated viruses with strong support (Figs 2A and 4B), as previously noted [27,44].

### Genomic and phylogenetic analysis of viruses other than EMCV

Genomic sequencing of the novel black rat adenovirus revealed a 28,894 bp genome, with 35 putative ORFs and 109 bp inverted terminal repeats. A phylogenetic analysis of the penton base demonstrated that it clustered with a long-tailed marmot (*Marmota caudata*) adenovirus from China with strong support, which together formed a clade with an Ord’s kangaroo rat (*Dipodomys ordii*) adenovirus from the southwestern United States, which similarly has a short genome relative to other mammalian adenoviruses (mastadenoviruses) [48] (S2 Fig). Alignment of DNA polymerase amino acid sequences against other adenoviruses showed a maximum identity of 59.1% to marmot adenovirus, demonstrating the black rat adenovirus is a novel mastadenovirus species [49].

Previously, we demonstrated MRV to be present within bat populations in North America [50] and nucleotide alignments of the coding regions of the 10 RNA segments (L1-L3; M1-M3; S1-S4) of the hispid cotton rat MRV isolate to big brown bat (*Eptesicus fuscus*) MRV isolate 17-Ef40 demonstrated a very close relationship in most segments (>99% nucleotide identity in L1, L2, L3, M1, M2, and S3). All segments showed >90% nucleotide identity to 17-Ef40, other than S1 (61.1%). Phylogenetic analysis of the σ1 protein encoded in S1 – the viral attachment protein used for cell entry and the main antigen determining serotype specificity – demonstrated it clustered with serotype 2 (T2) human isolates (S3 Fig). Interestingly, the hispid cotton rat σ1 protein has an insertion in its stalk relative to other MRVs, making it the longest σ1 protein (494aa) identified to date. The functional significance of this elongation is unknown. Similar to the discovery of MRV in North American bats, the isolation of MRV from a hispid cotton rat suggests the distribution and diversity of MRV within rodent populations in the United States is likely also underappreciated and may represent a source of transmission to humans and other animals.

### *In vitro* fitness and host range of elephant EMCV isolate N-037–19

**Fig 5:**
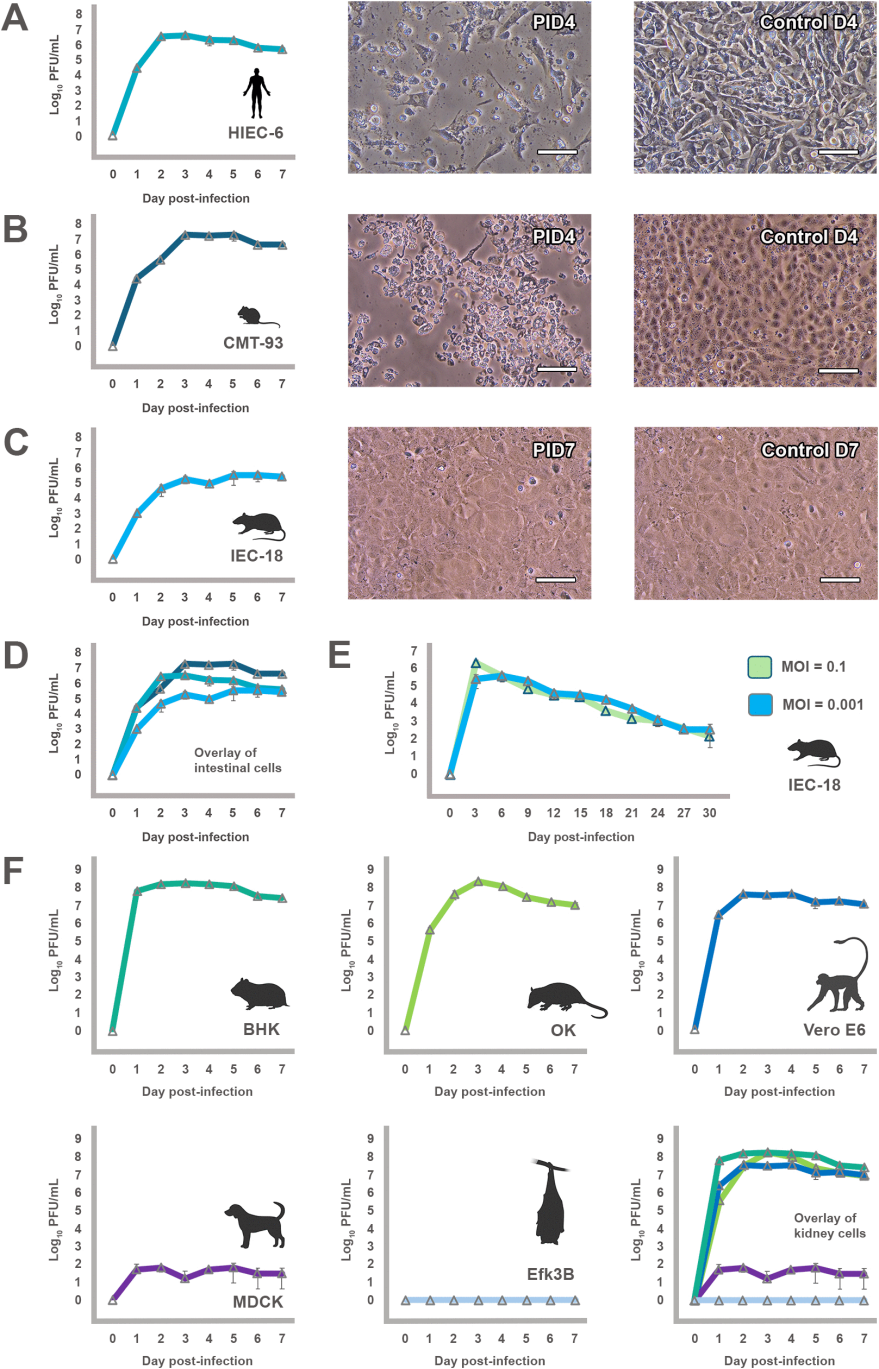
Replicative fitness of EMCV elephant isolate N-037-19 in intestinal and kidney cells of various hosts. **(A-E)** Multi-step growth curves and EMCV cytopathology in intestinal cells. Cells were infected at an MOI = 0.001 and harvested daily for 7 days. Cells shown are **(A)** HIEC-6 (human small intestinal enterocytes), **(B)** CMT-93 (House mouse large intestinal/rectal carcinoma cells), and **(C)** IEC-18 (Norway rat small intestinal [ileum] epithelial cells). Note EMCV infection in rat intestinal cells is non-cytolytic, in contrast to human and mouse cells. Scale bar = 100µm; **(D)** An overlay between the three cell lines highlights the reduced replication kinetics observed in rat intestinal cells over time; **(E)** Growth curves in IEC-18 cells infected with an MOI = 0.001 or 0.1 of EMCV over a 30-day period. Similar to the 7-day growth curve, EMCV infection in IEC-18 cells shows no cytopathology over the course of 30 days and infection is ultimately cleared from cells; **(F)** Multi-step growth curves in kidney cells from various hosts. Cell lines shown are BHK (Syrian golden hamster), OK (Virginia opossum), Vero E6 (African green monkey), MDCK (domestic dog), and Efk3B (big brown bat). Both BHK and Vero E6 cells, which are defective in interferon production [54,55], are routinely used in viral diagnostics for EMCV. Note a host restriction for EMCV in dog kidney and big brown bat kidney cells. An overlay of growth curves is shown to highlight the differences of titers in various kidney cell lines. Image was created, in part, using BioRender (Allison, A. [2026] https://BioRender.com/umgiypr).

Although the tissue tropism of EMCV in rodents *in vivo* has been well studied [11,13], little is known about the *in vitro* host range of contemporary EMCV field isolates, particularly regarding rodent species and cells of importance in allowing shedding into the environment (i.e., intestinal epithelial cells). Previous analysis of rat embryo, myocardial, glioma, and hepatoma cells *in vitro* has shown they are relatively insusceptible to infection [51,52]. We observed three notable differences when comparing intestinal cell infections in mice (CMT-93), rats (IEC-18), and humans (HIEC-6) (Fig 5A-5E): (i) no discernible cytopathic effects were observed in rat cells over the course of infection for either 7 or 30 days, while massive cell death was prominent in mouse or human cells within 3–4 days, (ii) maximum viral titers released from rat cells remained comparatively lower during the course of infection versus mouse and human cells, and (iii) virus was ultimately cleared from rat cells (i.e., no release of live virus could be detected from intact monolayers at days 33 and 36 post-infection). While the previous non-permissive status of rat cells to EMCV infection led to the speculation that rats are possibly dead-end hosts [52], the low to moderate viral production in association with a lack of cytopathology in intestinal cells observed here may be more suggestive of a co-evolutionary relationship between the virus and a reservoir species that has limited cell death and viral titers. The isolation of EMCV from intestinal tissues of black rats during the outbreak here (Fig 2A-2C) also supports this hypothesis, as do *in vivo* assessments in other *Rattus* species (i.e., Norway rats) where EMCV infection is devoid of clinical signs and macroscopic lesions, with concomitant fecal shedding of virus [13]. Further studies at determining the mechanisms of non-cytolytic infection and the limited replicative capacity in rat intestinal cells relative to other species is warranted.

To assess replication of the elephant virus (N-037–19) in various mammalian cell lines and gain a better understanding of its host range *in vitro*, kidney cells were chosen as a consistent cell type among host species, and also as a relevant tissue source for release into the environment as EMCV may be excreted in urine [10,53]. BHK cells, which are often used to propagate or isolate EMCV, had the greatest replicative capacity for EMCV of the mammalian cells tested, with maximum titers reaching log10^8.4^ PFU/mL after infection at a low MOI (Fig 5F). Most mammalian cells exhibited similar dynamics, including opossum kidney (OK) and Vero E6 cells, with rapid replication times and maximum titers reached within 1–3 d post-infection. However, as both BHK and Vero E6 cells are known to be deficient in interferon signaling [54,55], and EMCV is known to be highly sensitive to interferon [56], such cell lines are unlikely to represent accurate models of *in vivo* viral infection, but do show their utility for EMCV isolation in veterinary diagnostic laboratories. In contrast, a number of mammalian cells tested did not support a productive infection. While dogs have been reported to be infected with EMCV, including detection of the virus in kidneys [57], cultured dog kidney cells (MDCK) were essentially refractory to infection with EMCV N-037–19 at MOI = 0.001 (Fig 5F), 0.1, and 1.0. This finding corroborates historical reports on the lack of clinical signs in experimentally infected dogs [58], suggesting adaptation may be required for a productive infection. Similarly, inoculation of big brown bat kidney cell (Efk3B) monolayers did not result in a productive infection over the course of 7 days (Fig 5F) at various MOIs. However, EMCV N-037–19 replicated efficiently in Tb1Lu lung cells from Brazilian free-tailed bats, reaching a maximum titer of 10^7.6^ PFU/mL by day 2 post-infection. While the discrepancies in susceptibility between bat cell lines among various North American species is intriguing, genetic and phenotypic alterations in cultured cells such as loss of functional receptors or the utilization of alternative receptors may occur [59–61], thus these results require further study to determine their physiological significance. The role of bats as a potential reservoir for EMCV has been suggested, as EMCV has been detected in guano from eastern bent wing bats (*Miniopterus fuliginosus*) in Taiwan, South Korea, and Japan [62]. Bats as a source for EMCV in North America remains undetermined.

### Neutralizing antibody profiles in elephants and rhinos post-vaccination

**Fig 6:**
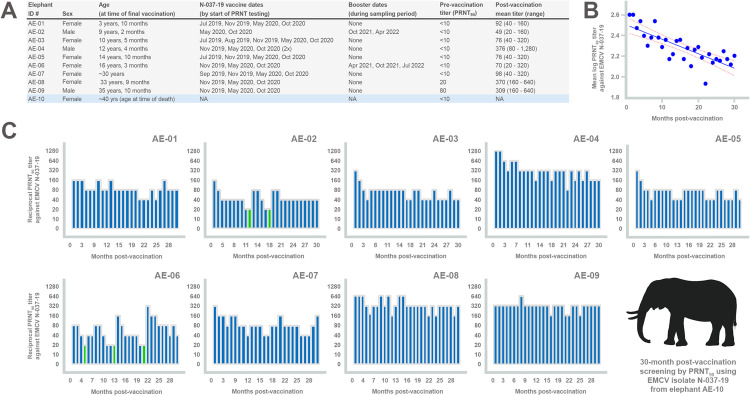
Neutralizing antibody responses to EMCV in autogenous vaccinated African elephants over a 2.5-year period. **(A)** List of elephants and their demographic data, along with vaccination schedules. Note all elephants received their final vaccine in a series in October 2020. Booster dates for elephants whose titers dropped to 1:20 (AE-02 and AE-06) post-October 2020 are indicated and are also highlighted as green bars in panel C. Note the two remaining oldest elephants – AE-08 and AE-09 – had pre-existing neutralizing antibodies against EMCV prior to vaccination. The deceased elephant (AE-10) did not have any evidence of previous infection. Post-vaccination mean titers indicate the average neutralizing antibody titer per number of sampling times, with corresponding titer ranges over the 30-month period; **(B)** Linear regression of mean log plaque-reduction neutralization test (PRNT_90_) titers versus months post-vaccination in elephants (slope = -0.014 [95% CI: -0.018 to -0.010], *R*^*2*^ = 0.62, *P*< 0.0001). Only elephants that did not receive a booster post-vaccination were included in the analysis; **(C)** Neutralizing antibody titers in the remaining nine elephants over the course of 30 months post-vaccination. Elephants are listed in order of ascending age. On average, elephants were bled at monthly intervals (mean = 31-35 days), although in some circumstances this may have been extended longer due to unforeseen circumstances as represented along the x-axis. Reciprocal antibody titers were determined using a 90% neutralization cut-off in the PRNT with the autogenous vaccine strain (N-037-19) derived from elephant AE-10 as the challenge virus. Image was created, in part, using BioRender (Allison, A. [2026] https://BioRender.com/51w9v48).

To discern the long-term duration and stability of neutralizing antibody responses post-vaccination, serum samples were collected approximately monthly from the remaining nine vaccinated African elephants over a period of 2.5 years (November 2019 – April 2023) (Fig 6). Note that neutralizing antibody responses during the vaccination series itself (i.e., between the first and last vaccination doses in the series) is shown in S4 Fig. Prior to vaccination, serum samples from all elephants were tested for pre-existing antibodies to EMCV, which also included testing archived serum samples from the 40-year-old female elephant that died from EMCV infection. Surprisingly, of the 10 elephants, two – AE-08 (a 33-year-old female) and AE-09 (and 35-year-old male) – had pre-existing anti-EMCV titers of 1:20 and 1:80 (based on a 90% neutralization positive cut-off), respectively (Fig 6A). The remaining elephants, including the deceased elephant AE-10, were negative for previous EMCV infection based on serology.

Of the nine vaccinated elephants, the two with pre-existing anti-EMCV antibodies (AE-08 and AE-09) demonstrated consistently high mean neutralizing antibody titers (1:370 and 1:309, respectively) for the complete duration of the study, suggesting such vigorous responses post-vaccination may have been a result of an anamnestic response due to previous exposure (Fig 6C). However, elephant AE-04, which lacked pre-existing antibodies, also demonstrated a strong neutralizing antibody response (mean titer of 1:376) consistently for a 30-month period, suggesting that vaccination alone with inactivated vaccines can result in high titer, long-term neutralizing antibodies. Although speculative, it is of interest that elephant AE-04 uniquely received back-to-back final vaccine doses in October 2020 (spaced two days apart as the first administration inadvertently resulted in only ~50% of the dose being given), which may have aided its strong neutralization titer. Similar to other studies [63,64], the magnitude of anti-EMCV neutralizing antibody responses varied considerably among individual animals of the same species, as some elephants had lower titers that tended to fluctuate. For instance, after the initial full vaccination series, elephant AE-06 had a neutralizing antibody titer of 1:80 (month 1), which over the course of the next several months dropped to 1:40, and then to 1:20 (Fig 6C). As a precaution, once titers dropped to 1:20, the elephants were administered a vaccine booster. In elephant AE-06, which received three boosters during the 2.5-year period, a consistent trend of an increase in titer post-booster, followed by a drop to pre-booster or near pre-booster levels, was observed (Fig 6C). Nevertheless, all elephants demonstrated detectable mean antibody titers ≥1:49 during the course of the study, with 7 of the 9 elephants not requiring a booster in 2.5 years to increase their titer, with linear regression demonstrating that, overall, neutralizing antibodies waned slowly in the elephants (slope = -0.014 [95% CI: -0.018 to -0.010], *R*^*2*^ = 0.62, *P*< 0.0001) (Fig 6B).

Vaccinated Southern white and Southern black rhinos (which are near threatened and critically endangered species, respectively) were also monitored to assess the efficacy of the vaccine. As with the results in the elephants, there was significant variation among individuals, with robust antibody responses that lasted for multiple years in certain animals (S5B Fig). In particular, white rhino SWR-05 exhibited a mean average titer of 1:3,151, although its testing was cut short to 16 months as it was transferred to another zoo. Similar to elephants AE-08 and AE-09, this rhino was shown to have pre-existing antibodies (1:20) to EMCV (S5A Fig), suggesting its exceptionally high titer was aided by previous exposure. White rhino SWR-04 also had a strong response to vaccination with an average monthly mean titer of 1:911 over 27 months, although its pre-vaccination titer was < 1:10. The majority of the remaining rhinos (5/7) maintained consistent neutralizing antibody titers, albeit lower than SWR-04 and SWR-05, and similar to the elephants, titers waned slowly throughout the study (slope = -0.025 [95%CI: -0.032 to -0.017], *R*^*2*^ = 0.63, *P* < 0.0001) (S5C Fig). Only a single rhino (white rhino SWR-03) exhibited a poor response to vaccination, with repeated boosters needed in order to maintain a mean monthly titer of 1:69. This rhino was also pregnant and gave birth during the serological testing period and was excluded from testing for 4 months post-birth (S5B Fig).

### Rescue and fitness of infectious clones

**Fig 7:**
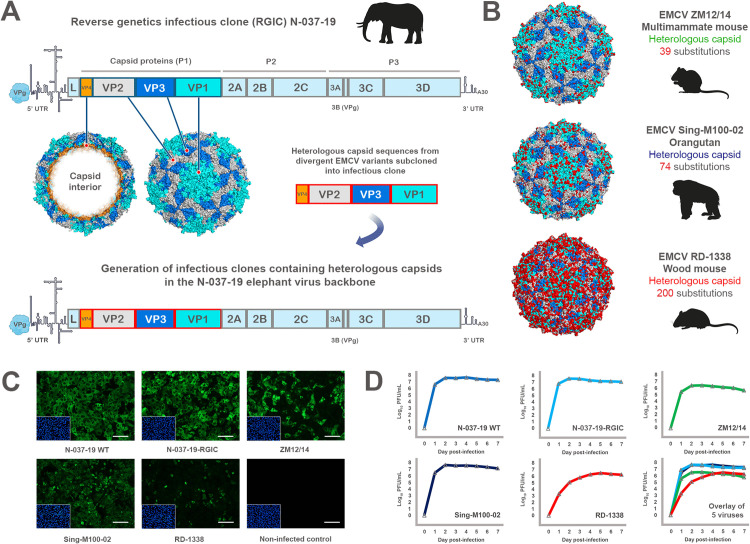
Development of a reverse genetics system for EMCV elephant isolate N-037-19 to evaluate cross-neutralization provided against divergent capsids after autogenous vaccination. **(A)** Strategy for generating infectious clones with heterologous capsids. The genome for EMCV N-037-19 is shown, which contains 5’ and 3’ UTRs flanking the polyprotein. The polyprotein is subdivided in three regions denoted as P1, P2, and P3, in addition to a leader (L) sequence preceding P1. P1 encodes the capsid proteins, specifically VP1, VP2, VP3, and VP4. Note that the outer capsid surface is composed of VP1-3 (with each protein color-coded for identity), while VP4 is found inside the capsid (shown in orange) and is therefore not exposed on the surface. The P2 and P3 regions encode proteins needed for replication and polyprotein processing and are thus not part of the virion structure; **(B)** Heterologous capsids generated using the elephant EMCV infectious clone. Three capsids representing EMCV-1 (ZM12/14; GenBank: LC585221), EMCV-2 (RD-1338; GenBank: JX257003), and putative EMCV-3 (Sing-M100-02; GenBank: KC310737) were generated, each with varying numbers of capsid substitutions relative to EMCV N-037-19 as shown. Non-synonymous amino acid changes are highlighted in red and mapped onto the Mengo capsid crystal structure (pdb_00002mev) using PyMOL; **(C)** Immunofluorescence assays of the three rescued viruses with heterologous capsids versus the elephant N-037-19 infectious clone and wild-type N-037-19 at 24 h post-infection in Vero E6 cells. Cells were infected at an MOI = 0.001 and fixed and stained with a swine anti-EMCV-1 polyclonal antibody and a goat anti-swine IgG Alexa Fluor 488 conjugated secondary antibody. Corresponding images of DAPI-stained nuclei are shown as an inset within each panel to visualize the confluent monolayer. Scale bar = 500µm; **(D)** Multi-step growth curves of the five viruses over seven days in Vero E6 cells. Note that while RD-1338 (EMCV-2) and ZM12/14 (EMCV-1) had reduced fitness relative to the wild-type and infectious clone N-037-19 viruses, Sing-M100-02 (EMCV-3) exhibited nearly identical growth curves. Image was created, in part, using BioRender (Allison, A. [2026] https://BioRender.com/tx8dnpi).

We rescued viruses containing heterologous capsid sequences from three divergent EMCVs that were cloned in place of the existing homologous N-037–19 capsid: (i) EMCV ZM12/14 (GenBank: LC585221), isolated from a multimammate mouse (*Mastomys natalensis*) in Zambia in 2012 and designated as serotype 1 [12], (ii) EMCV RD-1338 (GenBank: JX257003), isolated from a wood mouse (*Apodemus sylvaticus*) in Germany in 2005 and designated as the prototype serotype 2 [5]; and (iii) EMCV Sing-M100-02 (GenBank: KC310737), isolated from a Bornean (*Pongo pygmaeus*) and/or Sumatran (*P. abelii*) orangutan (exact species not listed) in Singapore in 2002 and tentatively designated as serotype 3 [27,65] (Figs 3 and 7A-7B). To discern the relative infectivity of the viruses, Vero E6 cells were infected at a low MOI (0.001) and fixed and stained at 24 h post-infection. Using an anti-EMCV-1 swine antibody for detection, widespread fluorescent staining was evident throughout the monolayers in the N-037–19 wild-type and infectious clone infected cultures, as well as for Sing-M100-02 (Fig 7C). In contrast, both ZM12/14 and RD-1338 showed restricted staining, suggesting more limited replication (Fig 7C).

Multi-step growth curves with the viruses over seven days showed a number of trends: (i) viral titers at 24 h post-infection correlated with the relative infectivity immunofluorescence results (i.e., limited fluorescence in the ZM12/14 and RD-1338 cultures was due to low titers) (Fig 7C-7D), (ii) viral titers between wild-type and infectious clone N-037–19 were nearly identical, suggesting the fitness of the clone was comparable to the wild-type virus, (iii) Sing-M100-02 (EMCV-3) produced viral titers almost indistinguishable from the N-037–19 viruses, and (iv) both ZM12/14 (EMCV-1) and RD-1338 (EMCV-2) exhibited a marked reduction in fitness relative to Sing-M100-02, which was particularly evident with RD-1338, reaching a maximum mean titer of log 10^6.5^ PFU/mL only after 5 days and was ~ 1500-fold lower at day 1 post-infection relative to Sing-M100-02 (Fig 7D). Comparison of the nucleotide and amino acid identity of available non-capsid sequences (i.e., omitting VP1–4) from the three heterologous viruses versus N-037–19 showed, surprisingly, that Sing-M100-02 was the most divergent (75.5% and 83.9% identity, versus 79.2% and 87.7% in ZM12/14 and 75.7% and 84.2% in RD-1338), suggesting that increased fitness relative to ZM12/14 and RD-1338 was not due to a higher degree of genetic compatibility with the background N-037–19 clone. However, without growth curves from the corresponding wild-type viruses of the three heterologous capsids, it is impossible to know if their lower relative fitness is a consequence of their chimeric configuration or not.

### Neutralization profiles against homologous viruses, various divergent wild-type EMCVs, and chimeric infectious clones

**Fig 8:**
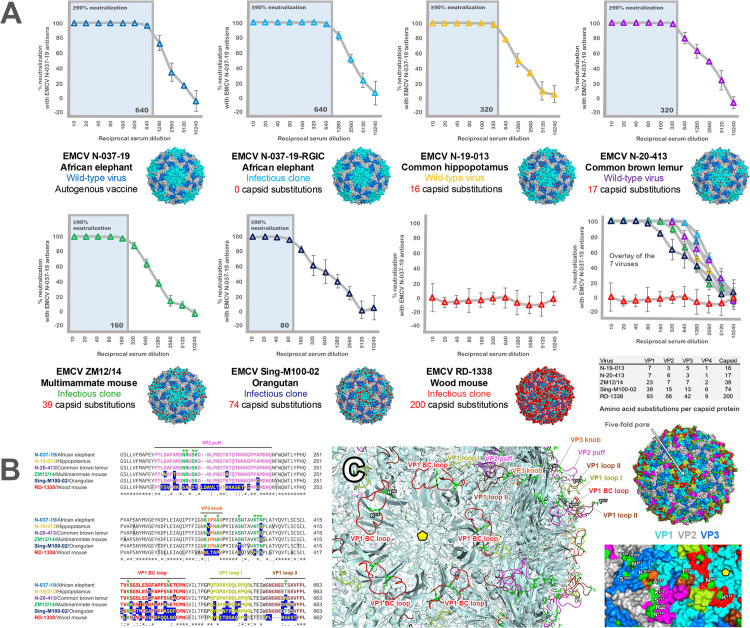
Neutralizing antibody titers of vaccinated elephants against wild-type viruses and infectious clones containing heterologous capsids. **(A)** Neutralizing antibody titers of vaccinated elephant antisera against (i) the homologous virus used as a vaccine (N-037-19) and its infectious clone, (ii) two recent isolates recovered from fatal zoological cases in Texas (hippopotamus) and south Florida (lemur), and (iii) the N-037-19 infectious clone containing capsids from divergent EMCV-1 (ZM12/14), EMCV-2 (RD-1338), and putative EMCV-3 (Sing-M100-02) isolates. Note that the viruses are ordered in terms of the increasing number of amino acid substitutions in their capsids relative to N-037-19, which were mapped onto the crystal structure of Mengo virus (pdb_00002mev) in PyMOL and shown in red; **(B)** Alignment of the known antigenic sites previously identified in EMCV [66,67] between the six viruses from panel A. Antigenic sites, based on neutralization escape mutations, include the VP2 surface puff (magenta), VP3 surface knob (orange), and 3 VP1 loop regions (BC loop [red], Loop I [lemon], and Loop 2 [brown]) [68]. Neutralization escape mutations are shown with a green diamond in the alignment. Differences between the viruses outside these sites are colored in gray; **(C)** Location of antigenic/neutralization escape mutation sites on the capsid. Antigenic sites are color-coded as in panel B. The cartoon of the capsid is shown on the left to highlight the elaborate loop structures that form the major antigenic sites. Escape mutations shown in the alignment are also highlighted in bright green in stick configuration and numbered in the surface map (bottom right). The five-fold pore – where viral RNA is extruded from the capsid – is highlighted in the capsid images with an arrow or yellow pentamer for orientation. The VP1, VP2, and VP3 proteins are highlighted in aqua, gray, and marine blue in the full capsid (upper right) and surface rendition (bottom right) only.

We first tested antisera from N-037–19 vaccinated elephants and rhinos against wild-type N-037–19 and the newly constructed infectious clone. Neutralization levels were very similar (endpoint 90% neutralization titer of 1:640), demonstrating that the infectious clone exhibited both replicative fitness (Fig 7D) and antigenicity (Fig 8A) comparable to the wild-type virus. Next, testing of the two EMCVs associated with the zoological deaths of a hippopotamus from Texas (N-19–013) and a common brown lemur from south Florida (N-20–413), which had 16 and 17 amino acid substitutions in their capsids, respectively, showed a slight drop in 90% neutralization relative to the homologous viruses (1:640–1:320), although both the lemur and hippopotamus viruses were still neutralized ≥50% at a 1:1,280 dilution (Fig 8A). As noted above, these two viruses clustered together outside the major EMCV-1 clade that contained the outbreak viruses and thus represent another EMCV-1 lineage that exists within the southeastern United States that zoological animals could readily encounter (Fig 3). Although the elephants were vaccinated twice with the hippopotamus-derived virus vaccine initially (April and May 2019), prior to getting a full series of autogenous vaccine doses against the elephant N-037–19 virus (between May 2019 and October 2020), neutralization titers (measured post-October 2020) were slightly higher against the elephant virus (1:640) than the hippopotamus virus (1:320) (Fig 8A).

To determine how well autogenous vaccine-induced antibodies neutralized more divergent viruses, including different known or tentative serotypes, we next examined the elephant infectious clone containing the heterologous capsids of EMCV ZM12/14 (EMCV-1), EMCV RD-1338 (EMCV-2), and EMCV Sing-M100-02 (EMCV-3). Overall, the level of neutralization corresponded to the number of amino acid substitutions in the capsid, with neutralization titers of 1:160 against ZM12/14 (39 substitutions), 1:80 against Sing-M100-02 (74 substitutions), and 0 against RD-1338 (200 substitutions) (Fig 8A). Other than RD-1338, all viruses showed decreasing levels of neutralization with dilution of N-037–19 vaccine antisera. RD-1338 did not exhibit neutralization at any dilution of N-037–19 vaccine antisera.

To better understand how amino acid changes in the capsid of the various viruses may have affected the neutralization profiles against the N-037–19 vaccine-induced antibodies, the capsid sequences of the six viruses were aligned together, and differences were then mapped to known antigenic sites/monoclonal antibody neutralization escape epitopes previously identified in the Mengo virus (EMCV-1) capsid [66,67] (Fig 8B). Thirteen neutralization escape mutations (shown as green triangles in Fig 8B) mapped to four main antigenic sites that are surface-exposed on the capsid: (i) VP2 “puff” region, (ii) VP3 “knob” region, (iii) a VP1 loop located between β-strands B and C (termed BC loop), and (iv) two loops that are in close proximity to the BC loop denoted VP1 loops I and II [3,68] (Fig 8C). Between Mengo virus and N-037–19, 12/13 of these neutralization escape sites were the same residue, with 401^N^ in N-037–19 and 401^Q^ in Mengo virus being the only difference. When comparing the five viruses against N-037–19, the number of mutations that occurred at these neutralization escape sites was 1/13 (hippopotamus), 2/13 (lemur, multimammate mouse, orangutan), and 11/13 (wood mouse). When comparing the number of substitutions within the entire four antigenic sites (color-coded in the alignment and surface map in Fig 8B-8C), amino acid changes observed were 3/94 (hippopotamus), 5/94 (lemur), 16/94 (multimammate mouse), 21/94 (orangutan), and 61/94 (wood mouse). Note that while Sing-M100-02 contained a number of substitutions in VP1 antigenic sites, there was limited changes in the VP2 puff or VP3 knob (Fig 8B), which may, in part, explain its neutralization by N-037–19 antisera (Fig 8A). In the case of the wood mouse, the 61 amino acid substitutions also included a 2-amino acid insertion in the VP2 puff and a 3-amino acid deletion in VP1 loop II based on alignments (Fig 8B), suggesting major structural changes to these antigenic sites in the EMCV-2 capsid, reinforcing the lack of any neutralization by N-037–19 antisera.

## Discussion

We describe an EMCV outbreak that occurred at a zoological institution in central Florida. In an attempt to gain a better understanding of the epidemiology and evolutionary genetics of EMCV, particularly in the southeastern United States where virus activity is prevalent yet understudied, we conducted the most comprehensive comparative phylogenomic analysis of virus isolates from fatal animal cases and local rodent species to date. We also investigated the duration and magnitude of neutralizing antibody responses in elephants and rhinos after autogenous vaccination, monitoring animals monthly for multiple years, thereby providing a unique fine-scale, long-term profile of responses to autogenous vaccination, which had not been previously assessed. We also determined if autogenous vaccine-induced antibodies were cross-protective against various divergent EMCV genotypes and novel serotypes, using an infectious clone derived from the elephant isolate, further illuminating the antigenicity of variant EMCVs found in nature and clarifying serotype diversity.

Although the full spectrum of the host range for EMCV remains unclear, many mammalian species appear to be susceptible to infection and clinical disease [15,20,65]. The course of disease may vary significantly among different mammal species, as well as with individuals of the same species, suggesting that many factors including age, overall health, immunocompetency, infectious dose, and virus strain variation play a role in host pathogenesis [20,21,69]. However, certain species, particularly African savanna elephants, have been noted to be highly susceptible to disease, with most fatalities being peracute, involving little or no premonitory signs prior to death [23]. This was also the case shown here, where a 40-year-old female African elephant – the index case of the zoo outbreak – was observed to be behaving normally by zoo personnel the day prior to being found dead, suggesting a sudden death with no overt signs of trauma or a struggle. EMCV has been shown to be predominantly cardiotropic in elephants [22–24,28,31], as was the situation here, as the elephant had severe necrotizing myocarditis and extensive epicardial hemorrhages, with virus being isolated from all tissue sections taken from the heart.

Interestingly, the heart lesions and concomitant clinical signs in the zoo animals followed two distinctive patterns. In the elephant and babirusa (non-primate cases), their deaths appeared to be largely peracute. This clinical presentation of a more or less sudden death was accompanied with severe epicardial and myocardial hemorrhages and peracute to acute myocardial necrosis with fulminant neutrophilic myocarditis, without overt signs of congestive heart failure. Conversely, the primate cases (mandrills and lion-tailed macaque) lacked the dramatic hemorrhages in the heart, with less acute myocardial necrosis, but with more myofiber degeneration and fragmentation. These lesions, in turn, were associated with clinical and gross findings that supported congestive heart failure, such as thoracic, pericardial, and abdominal effusion, along with pulmonary edema, with all primate cases exhibiting some degree of lethargy prior to death. The basis for these differences, and the dramatic differences in infection between non-human primates and humans, is currently not understood.

While elephants are thought to be highly susceptible to EMCV-induced disease, retrospective testing of pre-outbreak sera from the remaining nine elephants demonstrated that two elephants had neutralizing antibody titers to EMCV. While there is a possibility that these elephants were exposed to a related, yet undescribed cardiovirus that results in strong levels of cross-reactivity to EMCV in neutralization tests, it is likely suggestive that zoo animals may be exposed to EMCV more frequently than believed and that infection in elephants may not always result in severe clinical disease, as has been observed previously in some cases [32]. Both EMCV-seropositive elephants were the remaining oldest elephants and originated from other zoos outside Florida, so it is unclear when and where they were infected. However, surveillance in the local rodent population demonstrated that the virus can still be found at low levels during non-epizootic periods, which could theoretically result in concomitant low levels of exposure in zoo animals.

Despite many fatal outbreaks of EMCV within zoological parks in the United States within the last 50 years [16,19,22,23,63], the specific species of rodent reservoir and/or amplifying hosts responsible for spreading the virus to zoo animals have not been identified. Additionally, no viruses from outbreaks in zoological parks in the United States have been genetically characterized, leading to a lack of understanding of the diversity of EMCV that exists in North America and those viruses associated with animal deaths. Currently, the number of complete genome or full polyprotein sequences of field isolates of EMCV from the United States that are available in public databases is severely limited in comparison to Asia or Europe, with the last sequence being deposited in GenBank 22 years ago (i.e., EMCV isolate 30/87 recovered from Minnesota swine; GenBank accession AY296731), which consequently is the EMCV isolate most closely related to the central Florida outbreak viruses.

Within the southeastern United States, where EMCV zoo outbreaks tend to predominate [16,19,23], invasive non-native rat species – black and Norway rats – are often cited as a potential source of outbreaks based on circumstantial increases in rodent sightings preceding animal deaths. Our virus surveillance in the local rodent population, which included isolation of viruses from rodents during the outbreak itself, as well as post-outbreak, demonstrated black rats were the species from which the majority of rodent EMCV isolates were recovered and that these were phylogenetically linked to the viruses isolated from the zoo animals. However, in addition to black rats, EMCV was also isolated from three species of native rodents: Eastern woodrats, cotton mice, and hispid cotton rats. Collectively, all the rodent and zoo animal viruses were phylogenetically related to one another, suggesting a single introduction.

Based on these surveillance results, two basic hypotheses on the source of this particular outbreak can be proposed. One possible scenario is that EMCV is maintained normally in wild, native rodents (e.g., eastern woodrats, cotton mice, and/or hispid cotton rats) and that black rats only become infected once they enter new territory already occupied by native species. Black rats have previously been suggested to be indicators of virus activity, rather than true reservoirs, due to their relative resistance to infection [70,71], and are well known for their dispersal abilities and large capacity to colonize new environments, owing in part to their unique climbing and constant exploratory behavior [72]. The high number of black rats that may occur when a population encroaches upon new territory likely facilitates virus activity to higher levels by acting as a large pool of amplifying hosts, which ultimately increases the chances of viral spread to zoo animals. Of particular interest was our isolation of EMCV from a hispid cotton rat – which was phylogenetically related to the outbreak viruses – on park grounds two years post-outbreak, demonstrating the virus was still present in the local native population of rodents in the concomitant absence of noted disease in zoo animals during that time. That EMCV has previously been associated with hispid cotton rats in Florida [15], coupled with our detection here, is suggestive that this species should be investigated more thoroughly as a potential reservoir for EMCV in Florida and the southeastern United States.

EMCV may also be normally maintained in black rats in nature, and our isolation of the virus in native species simply represents spillover of EMCV once black rats encroached into the park. The isolation of EMCV from black rats after a zoological outbreak in France, also involving the death of an elephant [28,43], demonstrates that black rats are involved in the spread of the virus to zoo animals on multiple continents. As EMCV-1 has a worldwide distribution, this is suggestive that the geographical spread of the virus has been associated with the transcontinental movement of infected animals (i.e., swine) via trade, and/or with a species of rodent that is cosmopolitan in nature, of which only three species meet that criteria (i.e., black rat, Norway rat, and house mouse) [73]. Black rat encroachment into new territory – such as zoos – can often be associated with their displacement from their normal habitats due to disturbances, such as construction projects [74], which consequently were ongoing in the vicinity of the park at the time of the outbreak. Clearly, more emphasis on identifying the virus in rodent species during non-epizootic periods will be helpful in discerning its epidemiology. As EMCV will undoubtedly continue to cause outbreaks among zoos in the United States (and worldwide), identifying the source of the virus to potentially mitigate its spread, or at the very least better understand the epidemiology of viral outbreaks, is prudent.

Commercial killed vaccines for EMCV, once used widely in the United States to protect weanling swine from disease, are currently no longer in production, owing, in part, to the recognition of porcine reproductive and respiratory syndrome virus as a primary pathogen in swine outbreaks thought to be induced by EMCV [75]. Previously, a modified, live vaccine derived against Mengo virus (vMC_24_), which was attenuated via a shortened polyC tract, has been shown to be highly immunogenic and protective in macaques, baboons, and domestic swine, with virus being cleared rapidly [76]. A derivation of this vaccine (vMC_0_), with the polyC tract completely removed, previously showed promising results in zoo animals, with high neutralizing antibody titer responses in certain primate species, although in some animals there were mixed responses [63]. However, neither the vMC_24_ nor vMC_0_ vaccines are commercially available currently, and most zoos – usually in the response to animal deaths in their collection – have reverted to generating killed autogenous vaccines from viruses that have been isolated from their zoo animals, as was the case here. Killed vaccines are generally considered less effective than modified live vaccines due to potentially less robust antibody responses, the need for multiple boosters, and the question of whether long-term protection over the course of years (or the life of the animal) is afforded [76]. While autogenous vaccines against EMCV have been used extensively in the past, longitudinal studies to determine their long-term efficacy have not been evaluated previously.

After the death of the elephant from EMCV, we monitored the remaining nine elephants in the herd on a near monthly basis for 2.5 years. The majority did not require a booster after their vaccination series, as antibody titers did not drop below 1:40, and overall the waning of titers was slow, suggesting long-term protection. While animal challenge experiments are the prototypical format to examine the efficacy of a vaccine, such studies with the elephants and rhinos here are clearly not feasible. However, it has been demonstrated that titers ≥1:20 are protective in mice after challenge [76], and in one study, inactivated vaccine-induced antibodies were protective in African elephants subsequently challenged with EMCV [32]. These results suggest that killed autogenous vaccines – which in most circumstances are the only current option available to zoos for protecting their animals – can nevertheless induce long-lived, and in some cases, high-titer neutralizing antibodies that are likely protective against EMCV-related disease in elephants. Within the past few years, mRNA-based vaccines have been assessed for protecting elephants from elephant endotheliotropic herpesviruses [77], another serious pathogen of zoological elephants, and innovative mRNA-based strategies for non-enveloped viruses, such as picornaviruses (i.e., enterovirus D68), have been developed [78]. In the interim, before the potential development of new vaccine formats for EMCV, or the commercialization of existing live viral vaccines, these results provide some clarity on the magnitude of neutralizing antibody responses and the long-term efficacy of inactivated autogenous vaccines against EMCV in certain susceptible species.

Historically, genetic variants of EMCV found worldwide have been shown to retain antigenic cross-reactivity in neutralization tests and have thus been considered to represent a single serotype (EMCV-1), although they may differ in hemagglutinating activity [10]. However, a highly divergent EMCV strain (RD-1338) isolated from a captive wood mouse in Germany was shown to represent a second serotype (EMCV-2), as it was not neutralized by EMCV-1 hyperimmune sera [5]. Recently, other EMCV-2 variants have been detected extensively in wild hazel dormice (*Muscardinus avellanarius*) in England [79]. The phylogenetic clustering of rodent viruses from Vietnam, Thailand, and Singapore with the European viruses in the capsid phylogeny here suggests EMCV-2 may be widely distributed throughout Eurasia [45,46]. A third potential serotype (EMCV-3), comprising strains Sing-M100-02 and Sing-M105-02 isolated from captive orangutans in Singapore, has been suggested based on its phylogenetic divergence, although no comprehensive serological testing has been performed to confirm this tentative new serotype designation [27,65]. Newly identified divergent viruses from rats in Vietnam and China [47], have also been proposed to be novel serotypes (EMCV-4 and EMCV-5, respectively) [https://www.picornaviridae.com], but similar to EMCV-3, are currently based on genetic and/or phylogenetic divergence without serological testing.

To determine how well zoo animals vaccinated with an autogenous EMCV-1 vaccine are theoretically protected against heterologous viruses which have varying degrees of divergence in their capsid sequences, we tested the ability of vaccine-derived antisera from elephants to neutralize recent divergent wild-type viruses isolated from zoological outbreaks in Texas and Florida, in addition to reverse genetics-derived capsids of geographically and genetically disparate viruses, including EMCV-2 and EMCV-3. Our results corroborated previous findings regarding EMCV-2 isolate RD-1338 [5], in which expression of its capsid in association with the background elephant virus infectious clone resulted in no neutralization using antisera derived against the elephant virus (N-037–19; EMCV-1). In contrast, isolate Sing-M100-02 (EMCV-3) was still completely neutralized by elephant virus antisera, albeit at lower levels than other EMCV-1 isolates tested. As serotype designation is defined by the inability to be completely neutralized by antibodies raised against another serotype [80], the complete neutralization of EMCV-3 capsids with EMCV-1 antisera suggests it is not a novel serotype but rather is a divergent EMCV-1 variant. Our phylogenetic analysis also corroborates these findings, with Sing M100-02 clustering as an outgroup of the major EMCV-1 clade in the capsid phylogeny, distinct from the clade of EMCV-2 viruses and other divergent isolates which may represent additional serotypes. Although M100-02 had 74 amino acid substitutions in its capsid relative to our autogenous vaccine virus – including 21 substitutions in known antigenic sites – its neutralization (albeit 4-fold less than the homologous virus) suggests that autogenous vaccines may still provide protection against considerably divergent viruses.

As zoos may be reluctant to use autogenous vaccines derived at other institutions, fearing that they may not be protective against viruses circulating within their region, this study provides some assurance that even highly divergent EMCVs (of the same serotype) may be completely neutralized with autogenous vaccines. The utility of the development of a reverse genetics platform in which viral capsids can be readily exchanged is that it allows us to determine if vaccinated elephants will theoretically be protected against any EMCV variant in nature, even before the animals ever come into contact with such viruses. In addition, it will allow for future mapping of antigenic sites that are important in providing protection to zoo animals through mutagenesis studies and investigate discrepancies between *in vitro* and *in vivo* host ranges as noted here.

## Conclusions

Owing to the structural stability of the non-enveloped capsid, EMCV can persist for prolonged periods in the environment when conditions are favorable (e.g., the virus is surrounded by organic material such as soil, feces, or leaf litter in moist or humid environments), increasing the likelihood of contact with zoo animals through fecal- or urine-contaminated materials such as food, water, or bedding [13,21]. The potential long-term persistence of the virus in the environment, coupled with the ubiquitous presence of rodents and the severe and often fatal infection in many zoological species during epizootic conditions, as observed here, makes EMCV a constant threat to zoos and other animal collections. The socio-economic costs of EMCV outbreaks in zoos can be high due to the death (and/or quarantine) of animals, potential closure of exhibits, extensive rodent removal and abatement of nesting sites along with subsequent active rodent monitoring, in addition to the emotional toll on zoo personnel who took care of the deceased animals, in some instances for decades. In recent years, there have also been several EMCV outbreaks involving threatened or endangered species in managed care, thus putting additional strains on conservation efforts [81,82]. Understandably, these outbreaks and the loss of unique and endangered megafauna are a major concern to zoos and conservation programs worldwide. Although major gains have been made in recent years in better understanding the molecular mechanisms of EMCV pathogenesis [83–85], the ecology, evolution, and epidemiology of the virus in the United States remains vastly understudied, despite repeated zoological outbreaks. This has not only led to a lack of knowledge on the reservoir and amplifying host species that may be integral in precipitating outbreaks or maintaining the virus during non-epizootic periods, but also the genetic and antigenic diversity of EMCV in nature that exists, which may play a role in determining how well animals are protected from disease using currently available vaccines.

## Methods

### Ethics statement

All animal procedures (including blood sampling, vaccinations, and necropsy examinations) were conducted as part of the zoo’s clinical disease management, routine surveillance, and preventive medicine programs. As such, they did not require review or approval by the zoo’s Animal Care and Welfare Committee.

### Clinical and pathological examination of zoo animal cases during the outbreak

A pre-mortem clinical assessment was performed on the primates as they presented with rapidly progressing signs of congestive heart failure. The African elephant and the North Sulawesi babirusa were found dead with no overt preceding clinical signs or died very shortly after being found recumbent, respectively. Comprehensive post-mortem gross and microscopic examinations were performed on all six of the fatal animal cases that occurred during the outbreak (African elephant [one], mandrill [three], North Sulawesi babirusa [one], lion-tailed macaque [one]). Tissue samples from all major organ systems were collected and fixed in 10% neutral-buffered formalin (Thermo Fisher Scientific, Waltham, MA) and allowed to fix for 48–72 h. Fixed tissues were then routinely processed for histopathology, embedded in paraffin, sectioned at 4 µm, and the slides were stained with hematoxylin and eosin (H&E) for examination. Fresh tissue samples were shipped overnight to the University of Florida on ice packs for virus isolation.

### Virus isolation from zoo animals

For the six fatal animal cases, samples of heart and pooled tissues (liver, spleen, and/or brain) were tested for virus isolation. Tissue samples (~0.5 cm^3^) were mechanically homogenized in 650μL of Eagle’s minimum essential media (EMEM) supplemented with 5% fetal bovine serum (FBS) and 400 units/mL penicillin, 400μg/mL streptomycin, and 1μg/mL amphotericin B (4x antibiotics/antimycotics; Gibco, Thermo Fisher Scientific). Homogenized tissues were centrifuged at 6700 × *g* for 10 min and clarified supernatant (100 μL) was used to inoculate 2-day-old Vero E6 (African green monkey [*Chlorocebus aethiops*] kidney) and BHK-21 (Syrian golden hamster [*Mesocricetus auratus*] kidney) cells in a 12-well plate format. Both cell lines were purchased from the American Type Culture Collection (ATCC; Manassas, VA) and were maintained in EMEM with 5–10% FBS in a 5% CO_2_ atmosphere. Inoculated cultures were monitored daily for evidence of cytopathic effects (CPE). For wells exhibiting CPE, supernatant was harvested and aliquots frozen at -80°C. RNA was extracted from CPE-positive cultures using a QIAamp Viral RNA Mini kit (Qiagen; Valencia, CA) and identified as EMCV by RT-PCR using primers targeting a 414 nucleotide (nt) portion of the 3D polymerase gene. RT-PCR was performed using Improm-II RT (Promega; Madison, WI) and Phusion Plus DNA polymerase (Thermo Fisher Scientific) according to the manufacturer’s instructions. Primers and RT-PCR cycling conditions and parameters are available upon request. The African elephant prototype EMCV isolate (N-037–19) was passaged an additional time in Vero E6 cells to generate stock virus for use in plaque reduction neutralization tests and additional *in vitro* assays (see below).

### Virus isolation from wild rodents

To investigate the rodent species responsible for transmitting EMCV to the zoo animals, we instituted a comprehensive rodent control and removal program (in accordance with federal pest management regulations) in the vicinity of the enclosures of each of the fatal animal cases, in addition to other selected areas within the park, immediately following the initial EMCV animal deaths. From each collected rodent, intestinal samples (containing feces) and/or kidney samples were removed and sent to the Iowa State University Veterinary Diagnostic Laboratory in Ames, IA for EMCV qRT-PCR testing using a fluorogenic probe-based assay as previously described [86]. During the 2019 outbreak and up to post-outbreak Oct 2020, a subset of 40 qRT-PCR-positive samples were subsequently shipped to the University of Florida and tested for virus isolation in cell culture using the protocols listed above, with the addition of IEC-18 (Norway rat [*Rattus norvegicus*] ileum) cells (ATCC). Additionally, virus isolation was attempted on intestinal samples of 454 rodents harvested during post-outbreak surveillance from October 2020 to February 2021 (without prior qRT-PCR testing). For rodent samples from which EMCV (or other viruses) was isolated, host DNA was extracted from virus-positive tissue samples using a DNeasy Blood and Tissue Kit (Qiagen) for subsequent genetic identification of the species of rodent. PCR was performed using conserved primers targeting the vertebrate cytochrome B gene [87] and a Phusion High Fidelity or Phusion Plus DNA polymerase kit (Thermo Fisher Scientific) according to the manufacturer’s instructions. Amplicons were then Sanger sequenced to confirm the rodent species from which EMCV was isolated.

### RNA-Seq and viral genome assemblies

RNA was extracted from original tissue of EMCV-infected animals and/or low passage (P2) isolates from infected Vero E6 cell cultures using a RNeasy Plus Mini Kit with gDNA Eliminator spin columns (Qiagen) and/or treated on-column with RNase-free DNase (Qiagen). DNase-free RNA was then used to construct cDNA libraries using an Illumina Stranded Total RNA Prep with Ribo-Zero Plus (Illumina; San Diego, CA). A paired-end, 2 x 150 bp sequencing run of cDNA libraries was performed using an Illumina HiSeq 2000 platform. Paired-end FASTQ reads data were imported into Geneious Prime (Biomatters; Auckland, New Zealand) and Illumina adapters, low quality reads, and short reads (<30 bp) were removed using the bbduk plugin. EMCV reads were then assembled using the original RT-PCR amplicon as a starting template, followed by an iterative assembly method to construct the complete virus genome. For EMCV genomes in which the terminal non-coding regions could not be resolved sufficiently from the RNA-Seq data, 3’ and 5’ RACE was attempted using virus-specific primers and primers based on the FirstChoice RLM-RACE kit (Thermo Fisher Scientific). RACE amplicons were then Sanger sequenced. In an attempt to sequence through the polycytidine (polyC) tract in the 5’ UTR, direct RNA sequencing was also performed. Polyadenylated RNA was extracted from infected BHK cell cultures at 9 h post-infection at an MOI = 0.001 with a P1 isolate of N-037–19 (African elephant) using a PolyATtract mRNA Isolation System III (Promega; Madison, WI) and libraries were constructed using a Direct RNA Sequencing Kit (Oxford Nanopore Technologies [ONT], Oxford, United Kingdom) and a NEBNext Companion Module for ONT Ligation Sequencing (New England BioLabs, Ipswich, MA). Nanopore sequencing was performed using a GridION sequencer with FLO-MIN004RA flow cells. Basecalling with Dorado v0.9.1 was used under RNA super-accurate and 5-methylcytosine modification models. Samtools (v1.17) was used to convert basecalled BAM data into FASTQ data and analyzed as above.

For non-EMCV isolates recovered from rodents during EMCV surveillance, paired-end FASTQ reads from RNA-Seq or whole genome sequencing were assembled into contigs in Geneious using a medium-low sensitivity. Upon completion, contigs were translated in each frame and a local protein BLAST was performed using a virus database containing sequences downloaded from NCBI. Once a match to a known or related virus was confirmed, then a contig from which the BLAST hit originated was used to build the full genome by reiterative *de novo* assembly.

### Phylogenetic analysis

We performed phylogenetic analyses using a comprehensive set of EMCVs to examine the evolutionary relationships between our isolates from the fatal zoological animal cases and the local rodent population, as well as to other EMCV genotypes and serotypes found globally. Accordingly, we inferred phylogenetic trees based on (i) full genome (75 nucleotide sequences), (ii) polyprotein (L-VP4-VP2-VP3-VP1-2A-2B-2C-3A-3B-3C-3D) (75 amino acid sequences), (iii) capsid (VP4-VP2-VP3-VP1) (75 amino acid sequences), and (iv) 3D (RdRp) (74 nucleotide sequences). Both nucleotide and amino acid sequences were aligned using a combination of MAFFT [88] and Muscle [89] protocols. In the case of the full genome phylogeny, ambiguously aligned sites were removed using Gblocks (with parameters set to treat gap positions as only those positions where 50% or more of sequences have a gap) [90]. Phylogenetic trees of each data set were then inferred using the maximum likelihood (ML) method available in the PhyML package [91]. For protein phylogenies, we employed the LG + Γ model of amino acid substitution, while the GTR + Γ model was used for nucleotide trees (with relevant parameter values estimated from the data). Nodal support on each tree was estimated using SH-like branch supports. For non-EMCV isolates (adenovirus and mammalian orthoreovirus), we estimated amino acid phylogenies using the penton base (45 sequences) and sigma 1 (σ1) protein (114 sequences), respectively. In both cases, sequences were aligned using MAFFT, with Gblocks used to remove ambiguously aligned sites as above, and phylogenetic trees inferred using PhyML employing the LG + Γ model of amino acid substitution.

### Vaccine administration and serum collection from elephants and rhinoceroses

The outbreak began with the death of the African elephant in March 2019, at which time there was no EMCV vaccine in house to begin a vaccination program. However, in late 2018, an East African hippopotamus died from EMCV-induced necrotizing endomyocarditis at a zoo in Texas, after which an inactivated autogenous vaccine (Vaxxinova U.S. Inc., Worthington, MN) [64] was developed and was in current use at the Texas zoo at the time of the EMCV outbreak in Florida. To immediately begin vaccinating animals at the central Florida zoo (while an autogenous vaccine was being developed from the index elephant EMCV isolate), susceptible zoo animals – including African elephants, Southern white rhinoceroses (rhinos) (*Ceratotherium simum*), and Southern black rhinos (*Diceros bicornis*) (i.e., the specific animal species under longitudinal serological study here) – were vaccinated twice intramuscularly 2–3 weeks apart by hand injection either in the hind leg (elephants) or in neck region caudal to the ear (rhinos) between April and May 2019 with a single dose (2.0 mL) of the hippopotamus-derived vaccine. Starting in July 2019, animals then began a vaccination series using a single dose (1.0 mL) of a newly developed EMCV vaccine derived from the N-037–19 elephant EMCV isolate (Product #15098–03; Vaxxinova, U.S. Inc.). After the initial vaccine series was completed in May 2019, serum anti-EMCV titers were monitored monthly thereafter. While the vaccine manufacturer recommends booster doses every six months after an initial series of two vaccines spaced 2–4 weeks apart, vaccination schedules were instead guided by serology results since they provided up-to-date quantitative measurements of titers. For elephants, boosters were given when antibody titers fell to ≤1:20 based on plaque reduction neutralization tests (see below).

For serological testing of vaccine-induced neutralizing antibodies over time, blood was collected from the auricular veins in the ears of elephants and rhinos under voluntary control. Blood samples were placed in 8.5 mL BD Vacutainer serum separator tubes (SST)/blood collection tubes (Becton Dickinson, Franklin Lakes, NJ) and centrifuged at 1,327 x *g* for 10 min within 3 h from collection. Collected serum (after the final vaccination, along with pre-vaccination samples) was aliquoted into cryovials and frozen at -18 degrees °C until shipped on ice packs to the University of Florida for plaque reduction neutralization testing. Serum collected during the vaccination series itself was tested at the Animal Health Diagnostic Center, Ithaca, NY, using a serum neutralization microtiter assay.

### Plaque reduction neutralization testing of sera from vaccinated animals

To determine the efficacy of the autogenous vaccine in inducing neutralizing antibodies against EMCV, serum samples collected from vaccinated African elephants, white rhinos, and black rhinos were assayed by a plaque-reduction neutralization test (PRNT_90_). Briefly, serum samples were diluted 1:5 in EMEM, heat inactivated at 56 °C for 30 min, and 100μL was then mixed with an equal volume of MEM containing ~50–100 PFUs of EMCV N-037–19 (African elephant isolate) in a 96-well plate format. Samples (200μL) were incubated at 37 °C for 1 h, then allowed to adsorb to confluent Vero E6 cells in a 6-well plate format for 1 h with gentle rocking at 15 min intervals. After adsorption, 4 mL of a gum overlay containing 1% gum tragacanth/1X MEM supplemented with 4% FBS and 2x antibiotics/antimycotics was added to each well (Merck Millipore, Burlington, MA). Cultures were inactivated on day 2 post-adsorption with 10% buffered formalin and stained with 0.25% crystal violet (Thermo Fisher Scientific) for plaque visualization. Samples that were considered positive on the initial 1:10 screen (≥90% plaque reduction as compared to control levels) were serially diluted to determine their final endpoint titer. Titers <1:10 were considered negative.

### Statistical analysis

Data related to PRNT_90_ titers in elephants and rhinos were log transformed to normalize the data. A Repeated Measures One-Way Analysis of Variance (ANOVA) procedure was performed to assess the mean differences in PRNT_90_ titers over the months after vaccination in elephants, with alpha set at 0.05 for the data. An ANOVA with a mixed effect model was used for the rhino data. Dunnett’s multiple comparisons test was performed with alpha set at 0.001 for multiple comparisons. Mean difference and 99.9% confidence intervals were calculated, and significant differences in mean PRNT_90_ titer between the first month and the following months were determined. For correlative analyses, a linear regression model with least squares fit was used with log-transformed PRNT_90_ titer values to better approximate linearity between PRNT_90_ titers and months after vaccination. A p-value of 0.05 was considered significant. All statistical analyses were conducted in GraphPad Prism 10.

### Host range and *in vitro* fitness of the wild-type elephant EMCV

To examine the replicative fitness and host range of the wild-type elephant EMCV N-037–19 isolate *in vitro*, the following 9 cell lines were analyzed (chosen for relevance for virus isolation purposes, or as species [or related species] which have been shown to be infected in nature): (i) BHK; (ii) CMT-93 (house mouse [*Mus musculus*] large intestine/rectal carcinoma); (iii) Efk3B (big brown bat [*Eptesicus fuscus*] kidney); (iv) HIEC-6 (human small intestine enterocyte); (v) IEC-18; (vi) MDCK (domestic dog [*Canis lupus familiaris*] kidney); (vii) OK (Virginia opossum [*Didelphis virginiana*] kidney); (viii) Tb1Lu (Brazilian free-tailed bat [*Tadarida brasiliensis*] lung), and (ix) Vero E6 cells. Cell lines were purchased from ATCC or Kerafast (Boston, MA). HIEC-6 cells were grown in Opti-MEM supplemented with 10mM Glutamax (Gibco), 5% FBS, 100ng/µL epidermal growth factor (Corning, Corning, NY), and 1x antibiotics/antimycotics. All other cells lines were grown in EMEM or Dulbecco’s MEM (DMEM) supplemented with 5–10% FBS and 1x antibiotics/antimycotics.

For multi-step growth curve analysis in each cell line, confluent monolayers in a 12-well plate (3.8 cm^2^) format were infected with a low MOI (0.001 PFU/cell). Virus was allowed to absorb to cells for 1 h, inoculums were removed, and cells were washed 3x with sterile cell culture grade PBS and growth media was added. Wells were harvested at daily intervals for 7 days and frozen at -80°C until further processing. Additionally, for IEC-18 cells (which showed no CPE; see results section), growth curves were performed for 30 days at MOI of 0.001 and 0.1, with wells harvested at 3-day intervals. Experiments were performed in triplicate. To determine if IEC-18 cells were still producing any virus at the end of the experiment, day 30 cells were washed 3x, growth media was added, and wells were assayed again for virus production on days 33 and 36. For titrations, 10-fold serial dilutions of virus aliquots were allowed to adsorb at 37 °C for 1 h in a 6-well plate of Vero E6 cells with gentle rocking at 15 min intervals. The monolayer was then covered with a gum overlay and fixed and stained according to the protocol in the PRNT section above. Titers were expressed as log_10_ PFU/mL.

### Construction of a reverse genetics infectious clone

To construct an infectious clone for EMCV, a cDNA copy of the African elephant N-037–19 EMCV genome (with the cDNA-derived shortened polyC tract) was cloned into a pcDNA3.1(+) vector using *PmeI* sites to create pcDNA3.1-EMCV-N-037–19 (GenScript, Piscataway, NJ). To ensure the correct terminal ends of the genome were generated, the 5’ end of the genome was flanked with a hammerhead ribozyme site, which included a 14 nt 5’ end complementary sequence, and the 3’ end was flanked with a hepatitis delta virus (HDV) ribozyme site. Prior to the HDV ribozyme site, a poly-A tail of 30 nt was inserted, which is the mean average poly-A tail experimentally determined for EMCV [92]. To rescue live virus, pcDNA3.1-EMCV-N-037–19 was transfected into Vero E6 cells at 90% confluency in a 6-well format using 3.0μL of TransIT-LT1 reagent (Mirus Bio, Madison WI) per µg of plasmid. Once significant CPE was observed after transfection, supernatant was passed into new Vero E6 cells for generation of stock virus. Rescued virus was titered by 10-fold serial dilutions in a 6-well plate format via plaque assay as listed above.

### Assembly of infectious clones with alternate capsids and relative infectivity

To generate viruses containing the leader (L), P2 (2A, 2B, 2C), and P3 (3A, 3B, 3C, 3D) genomic sequences from the elephant virus, but with novel capsids (P1; VP4-VP2-VP3-VP1) from other EMCVs, capsid sequences were subcloned into the pcDNA3.1-EMCV-N-037–19 backbone plasmid by a modified Gibson assembly method. Chimeric viruses were constructed using a NEBuilder HiFi DNA Assembly Master Mix (New England Biolabs, Ipswich, MA) according to the manufacturer’s instructions. All capsid (VP4-VP2-VP3-VP1) sequences from alternate viruses were cloned into pcDNA3.1(+) (GenScript) and flanked by 5’ leader and 3’ 2A sequences of the elephant virus prior to being amplified for assembly into pcDNA3.1-EMCV-N-037–19. New chimeric clones were designated as backbone/insert (e.g., pcDNA3.1-EMCV-N-037–19/VP4231-RD-1338). Chimeric viruses were rescued in Vero E6 cells as noted above with all infectious clone stock viruses titrated by plaque assay and sequence confirmed.

To determine the relative infectivity levels of the chimeric viruses to each other and the original infectious clone and wild-type virus, Vero E6 cells were infected at an MOI = 0.001 and fixed at 24 h post-infection with 10% neutral-buffered formalin, washed 3x with PBS, and incubated with a 1:200 dilution of a swine anti-EMCV polyclonal antibody (301-MDV; National Veterinary Services Laboratory, Ames, IA) in permeabilization buffer (1X PBS, 0.5% bovine serum albumin, 0.5% Triton X-100) for 1 h while shaking. The wash steps were repeated, and the cells were then incubated with a 1:200 dilution of an AffiniPure goat anti-swine IgG (H + L) Alexa Fluor 488 conjugated antibody (Jackson ImmunoResearch, West Grove, PA) for 1 h while shaking, followed by a final wash series. Cells were then counterstained with DAPI (5 µg/mL; Thermo Fisher) for 3 min. Immunofluorescent images were captured using a Nikon Ts2-FL inverted microscope equipped with a DS-Fi3 digital camera with LED-DAPI and LED-FITC filters (Nikon Instruments; Melville, NY). Fitness of the viruses with heterologous capsids relative to the original elephant infectious clone and wild-type virus were then compared in Vero E6 cells and titrated using protocols mentioned above.

### Capsid modeling of amino acid substitutions

To spatially map amino acid differences between the capsids of various EMCVs, we used the structural model of the Mengo virus capsid solved at a 3.0 Å resolution (PDB ID: 00002mev) [3,68] using PyMOL (Schrodinger; New York, NY). In particular, we mapped mutations observed in the VP1, VP2, VP3, and VP4 capsid proteins of the chimeric viruses relative to the capsid of the homologous infectious clone of the African elephant virus N-037–19.

## Supporting information

S1 Fig Correlative EMCV qRT-PCR Ct values and virus isolation during the 28-day outbreak period. Ct values for the EMCV qRT-PCR of tissues with corresponding virus isolation results for the six fatal zoo animal cases, along with 24 rodents tested, are shown. Note Ct values are ordered from lowest to highest. Samples in which virus was isolated are shaded in gray. Rodents (rats/mice) in which virus was not isolated were not identified to species and are thus listed as “unspeciated rodent”. Note that while black rat R-017–19 was positive for EMCV by qRT-PCR based on cut-off values, EMCV was not isolated, but rather a novel adenovirus (black rat adenovirus; see S2 Fig). Image was created, in part, using BioRender (Allison, A. [2026] https://BioRender.com/a72h7dc).
(TIF)

S2 Fig Maximum likelihood amino acid phylogeny of the penton base of a novel adenovirus isolated from a black rat (*Rattus rattus*) during EMCV outbreak surveillance and its relationship to other mastadenoviruses. The evolutionary relationships of a novel adenovirus (R-017–19) – isolated during the EMCV outbreak in central Florida – were determined using viruses recovered from various mammalian hosts from North America, Australia, Europe, and Asia. The penton base is a pentameric protein found at each of the 12 vertices in the icosahedral adenovirus capsid and acts as a scaffold for the fiber attachment protein, as well as facilitating entry by binding to cell surface integrins after initial fiber binding [93]. The tree is mid-point rooted for clarity only, with horizontal branch lengths scaled according to the number of amino acid substitutions per site. SH-like branch supports >0.9 are shown. The new adenovirus is highlighted in blue bold and clusters with strong support to recently identified rodent adenoviruses from China and the United States. Viruses are labeled as: virus name/ scientific name of species of detection/ virus ID/ GenBank accession number/ state (where applicable)/ country/ isolation or detection date. Image was created, in part, using BioRender (Allison, A. [2026] https://BioRender.com/kkoskqs).
(TIF)

S3 Fig Maximum likelihood amino acid phylogeny of the σ1 protein of a mammalian orthoreovirus (MRV) isolated from a hispid cotton rat (*Sigmodon hispidus*) during post-outbreak EMCV surveillance and its relationship to other MRVs. The σ1 amino acid phylogeny was inferred using a comprehensive collection of MRVs from various mammalian species from Asia, Africa, Europe, and the Americas, along with those recovered from wastewater in Asia. σ1 is the viral capsid attachment protein that dictates host range and is the major antigenic protein that elicits host neutralizing antibody responses and determines serotype [94]. A close-up view of the clade containing the hispid cotton rat R-261–21 isolate (highlighted in blue bold) is shown in the inset. Big brown bat isolate 17-Ef40 from Pennsylvania, which shows high identity to R-261–21 in numerous RNA segments (see text for details) [50], is also highlighted. The tree is mid-point rooted for clarity only, and horizontal branch lengths are scaled according to the number of amino acid substitutions per site. SH-like branch supports >0.9 are shown, with only major clades highlighted. Viruses are labeled as: MRV serotype/ scientific name of host species/ virus ID/ GenBank accession number/ state (where applicable)/ country/ isolation or detection date/ protein. Four different serotypes of MRV have been reported and are shown in the phylogeny: T1 (prototype Lang), T2 (Jones), T3 (Dearing), and T4 (Ndelle) [94]. Note T4 Ndelle virus is not phylogenetically supported as being different from T3 viruses. Image was created, in part, using BioRender (Allison, A. [2026] https://BioRender.com/azxn3hy).
(TIF)

S4 Fig Pre-vaccination titers and interim titers during the vaccination series in elephants and rhinos as measured by a serum neutralization (SN) test. Vaccine titers are shown for (A) elephants and (B) rhinos. Antibody titer(s) determined between each vaccine dose are shown, unless not tested (NT). Note that two different vaccines were used in response to the outbreak: (i) an autogenous vaccine derived from an East African hippopotamus that died at a Texas zoo in late 2018 (Texas vaccine), which was used immediately after the central Florida outbreak began, followed by (ii) an autogenous vaccine derived from the index African elephant case that died from EMCV in central Florida in March 2019 (Florida vaccine). Vaccination with the Florida autogenous vaccine began in July 2019, three months after the Texas vaccine. The ‘First post vaccination plaque-reduction neutralization test (PRNT_90_) titer’ row in the elephants and rhinos (bottom row in A and B) corresponds to month 1 in Figs 6C and month 0 in S5B, respectively. Note that in the rhino data, some titers were determined by PRNT_90_ rather than the SN microtiter format, as denoted by an asterisk (*) in Florida vaccine dose #2. The post-vaccination serological results for the elephants and rhinos are shown in Figs 6C and S5B, respectively.
(TIF)

S5 Fig Neutralizing antibody responses to EMCV in autogenous vaccinated Southern black (SBR) and white rhinos (SWR) over a 2.5-year period. (A) List of rhinos and their demographic data, along with vaccination schedules. All rhinos received their final vaccine in a series between October 2020 and January 2021. Note pre-vaccination titer of SBR-01 (highlighted with an *) is a serum neutralization titer rather than a plaque-reduction neutralization test (PRNT_90_) titer (see S4 Fig); (B) Neutralizing antibody titers of seven rhinos over the course of 17–30 months post-vaccination. The first blood draw after final vaccination occurred within 0–4 days and, hence, the first post-vaccination month is recorded as “0”. On average, rhinos were bled at monthly intervals (mean = 31–41 days), although in some circumstances this may have been extended longer due to unforeseen circumstances as represented along the x-axis. Reciprocal antibody titers were determined using a 90% neutralization cut-off in the PRNT with the autogenous vaccine strain (N-037–19) derived from elephant AE-10 as the challenge virus. Note white rhino SWR-05 was transferred to another zoo at month 17 of the study; (C) Linear regression of mean log PRNT_90_ titers against months post-vaccination in rhinos (slope = -0.025 [95%CI: -0.032 to -0.017], *R*^*2*^ = 0.63, *P*< 0.0001). Rhino SWR-03, which received multiple boosters post-vaccination, was excluded from the analysis. For estimating post-vaccination mean titers, month 0 was excluded from the analysis. Rhino icon was obtained from VectorStock [95].
(TIF)

S1 Data Raw Data. Quantitative data for Figs 5, 6, 7, 8, and S5.
(XLSX)
