# Supplementary figures and images for: Zoological transmission of encephalomyocarditis virus in the United States: Virus evolution, host ecology, and capsid antigenicity derived from an outbreak
Source: PLoS Pathog. 2026 Feb 17;22(2):e1013861. doi: 10.1371/journal.ppat.1013861 (PMC12912594; doi:10.1371/journal.ppat.1013861)

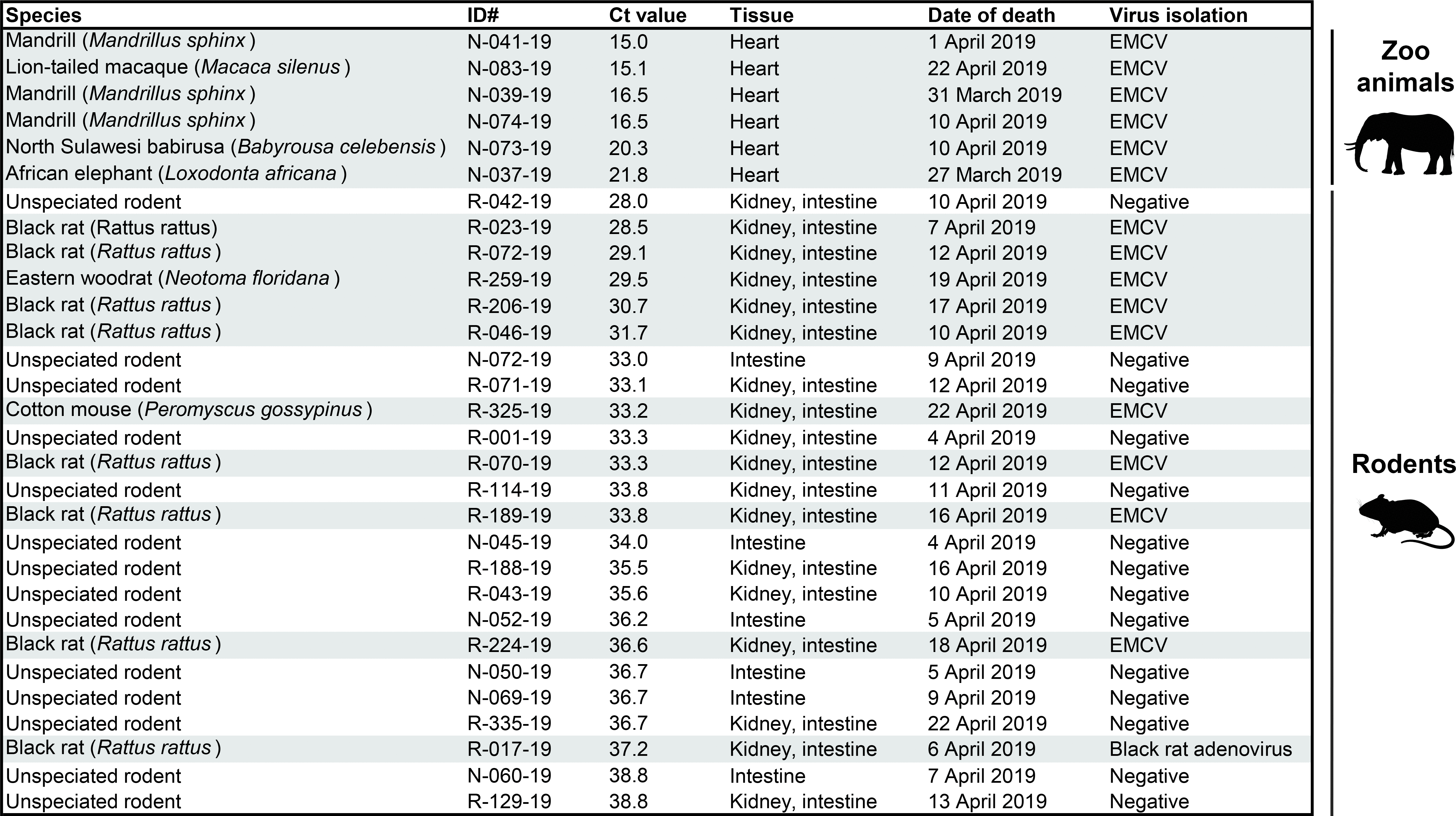

Supplement: S1 Fig — Ct values for the EMCV qRT-PCR of tissues with corresponding virus isolation results for the six fatal zoo animal cases, along with 24 rodents tested, are shown. Note Ct values are ordered from lowest to highest. Samples in which virus was isolated are shaded in gray. Rodents (rats/mice) in which virus was not isolated were not identified to species and are thus listed as “unspeciated rodent”. Note that while black rat R-017–19 was positive for EMCV by qRT-PCR based on cut-off values, EMCV was not isolated, but rather a novel adenovirus (black rat adenovirus; see S2 Fig). Image was created, in part, using BioRender (Allison, A. [2026] https://BioRender.com/a72h7dc). (TIF) [file ppat.1013861.s001.tif]

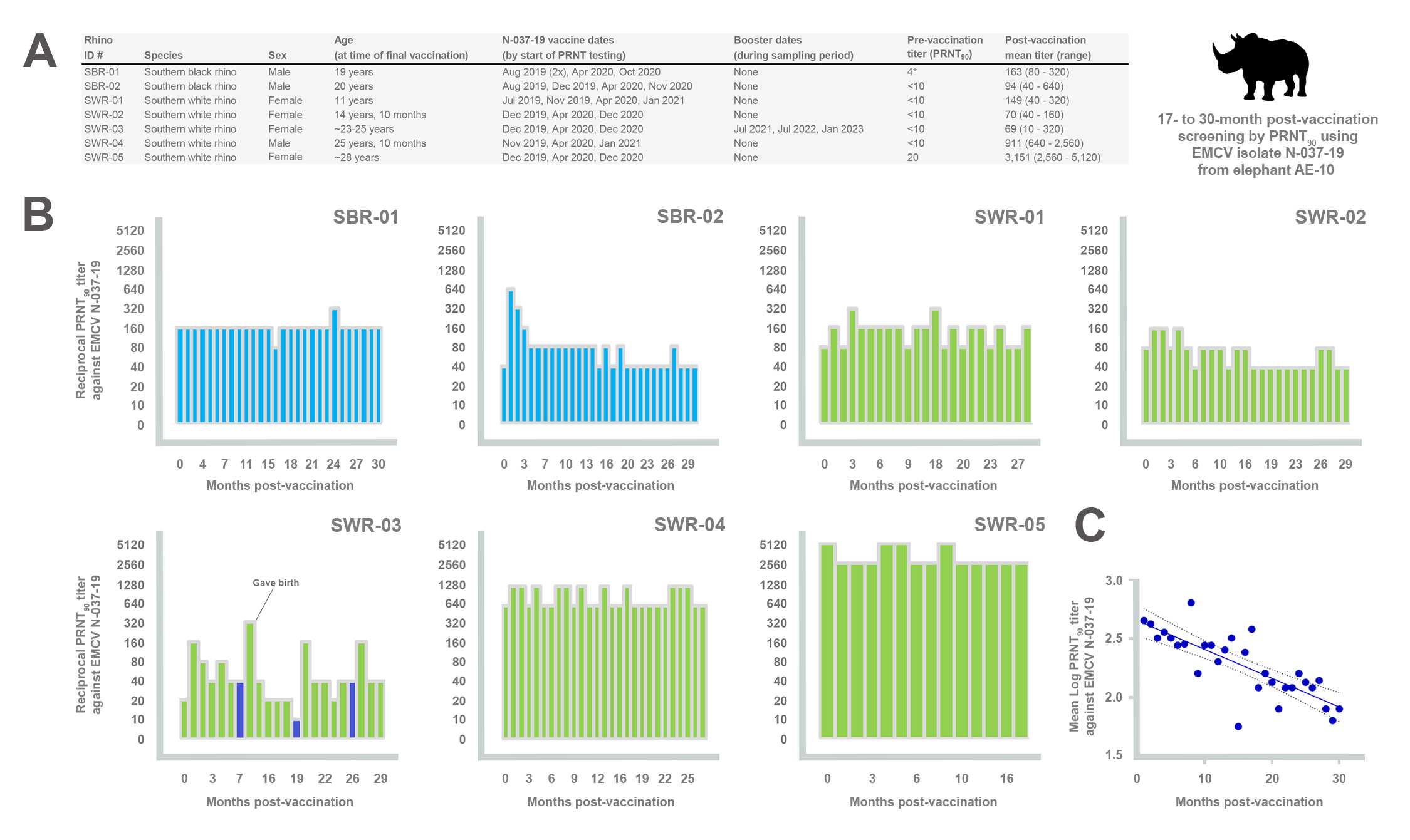

Supplement: S5 Fig — (A) List of rhinos and their demographic data, along with vaccination schedules. All rhinos received their final vaccine in a series between October 2020 and January 2021. Note pre-vaccination titer of SBR-01 (highlighted with an *) is a serum neutralization titer rather than a plaque-reduction neutralization test (PRNT90) titer (see S4 Fig); (B) Neutralizing antibody titers of seven rhinos over the course of 17–30 months post-vaccination. The first blood draw after final vaccination occurred within 0–4 days and, hence, the first post-vaccination month is recorded as “0”. On average, rhinos were bled at monthly intervals (mean = 31–41 days), although in some circumstances this may have been extended longer due to unforeseen circumstances as represented along the x-axis. Reciprocal antibody titers were determined using a 90% neutralization cut-off in the PRNT with the autogenous vaccine strain (N-037–19) derived from elephant AE-10 as the challenge virus. Note white rhino SWR-05 was transferred to another zoo at month 17 of the study; (C) Linear regression of mean log PRNT90 titers against months post-vaccination in rhinos (slope = -0.025 [95%CI: -0.032 to -0.017], R2 = 0.63, P< 0.0001). Rhino SWR-03, which received multiple boosters post-vaccination, was excluded from the analysis. For estimating post-vaccination mean titers, month 0 was excluded from the analysis. Rhino icon was obtained from VectorStock [95]. (TIF) [file ppat.1013861.s005.tif]
